# PhI(OAc)_2_-Promoted 1,2-Transfer Reaction between 1,1-Disubstituted Allylic Alcohols and Thiophenols

**DOI:** 10.3390/molecules29133112

**Published:** 2024-06-29

**Authors:** Guozhe Guo, Wenduo Li, Jingjing Zheng, Aping Liu, Qi Zhang, Yatao Wang

**Affiliations:** Gansu Key Laboratory of Efficient Utilization of Oil and Gas Resources, College of Petroleum and Chemical Engineering, Longdong University, Qingyang 745000, China; lwd0522@126.com (W.L.); 2019118113@nwnu.edu.cn (J.Z.); frdrld@126.com (A.L.); guoguozhe2008@sina.cn (Q.Z.); ziyu0023@163.com (Y.W.)

**Keywords:** allylic alcohols, thiophenols, β-carbonyl sulfides, PhI(OAc)_2_, 1,2-transfer reaction

## Abstract

The PhI(OAc)_2_-promoted 1,2-transfer reaction between allylic alcohols and thiophenols, conducted in an argon atmosphere, has proven to be effective in producing β-carbonyl sulfides from 1,1-disubstituted allylic alcohols in high yields. This method offers a fast and efficient way to synthesize β-carbonyl sulfides, which are valuable intermediates in organic synthesis. This discussion focuses on the effects of the oxidizer, temperature, and solvent on the reaction. A proposed tentative mechanism for this reaction is also discussed.

## 1. Introduction

**Scheme 1:**
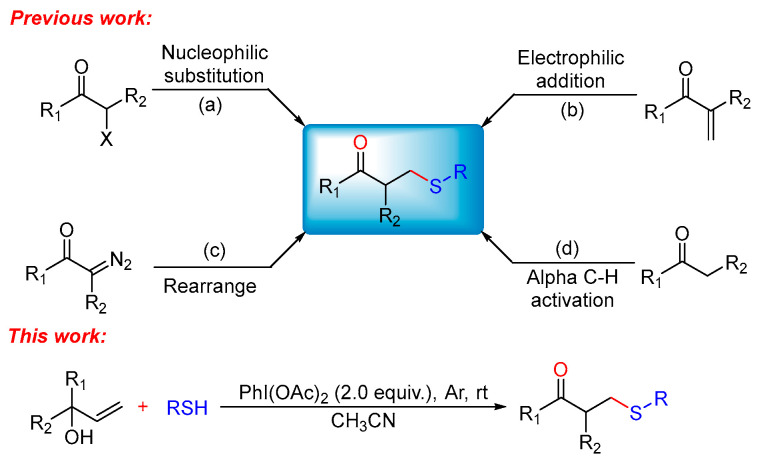
Synthesis of β-carbonyl sulfides.

Sulfur-containing structural motifs play a prominent role in the construction of protein [1,2,3,4], pharmaceutical [5,6,7,8,9], biological [10,11,12], and material molecules [13,14,15,16,17,18]. In particular, β-carbonyl sulfides have attracted much interest because not only are they easy building blocks that can be used to obtain organic compounds [19,20,21], but also, they are privileged structural scaffolds extensively present in drugs [22,23,24], beverages [25] and foods [26]. Thus, the construction of β-carbonyl sulfides has drawn more attention. The conventional procedures aimed toward the synthesis of β-carbonyl sulfides include (Scheme 1) (a) nucleophilic substitution [27,28], (b) electrophilic addition [29,30,31,32], (c) rearrangement reaction [33], and (d) alpha C-H activation reaction [34]. Although these approaches to building β-carbonyl sulfides are accessible, the developed methods often have some drawbacks, such as low selectivity, critical reaction conditions, and expensive transition metal catalysts. Thus, the improvement of novel synthetic techniques for the synthesis of specific structures remains a difficult task for organic chemists.

The functionalization of allyl alcohol follows the principle of atom economy, as it does not lead to the loss of atoms during the reaction. Specifically, when 1,1-diaryl allyl alcohol compounds are functionalized, rearrangement often occurs, i.e., an aryl group migrated from position 1 migrating to position 2, resulting in the formation of α-aryl-β-substituted carbonyl compounds. This has attracted increasing attention from organic chemists. Currently, various methods including light, electricity, peroxides, and transition metals can facilitate the smooth progression of the 1,2-aryl migration reaction in 1,1-diaryl allyl alcohols. In 2013, the Tu group developed [35] a copper-catalyzed trifluoromethyl of 1,1-diaryl allylic alcohols via 1,2-aryl migration. In 2014, the Ji group reported [36] the phosphonation of 1, 1-diaryl allylic alcohols via 1,2-aryl migration. In 2018, the Cai group reported [37] a light-catalyzed sulfoxide methyl of 1, 1-diaryl allylic alcohols synthesized via 1,2-aryl migration. In 2019, the Lei group reported [38] the synthesis of β-trifluoromethyl ketone under electrochemical conditions. In 2015, the Han group [39] reported the DTBP-catalyzed alkylation of 1, 1-diaryl allylic alcohols, which involves a 1,2-aryl migration process. Previously, our group reported [40] that K_2_S_2_O_8_ promoted dehydrative cross-coupling between 1,1-disubstituted allylic alcohols and thiophenols/thiols, and allyl sulfides were obtained. Herein, we report a study on the PhI(OAc)_2_-promoted 1,2-transfer reaction between allylic alcohols and thiophenols, which provided atom-economic and metal catalyst-free conditions for the synthesis of β-carbonyl sulfides.

## 2. Results

Initially, we chose 1,1-diphenylprop-2-en-1-ol (**1a**) and 4-methylthiophenol (**2a**) as the model substrates to optimize the reaction conditions, as shown in Table 1. We found that when the reaction was carried out in the presence of PhI(OAc)_2_ at room temperature in an argon atmosphere for 24 h in CH_3_CN, the β-carbonyl sulfide **3aa** was obtained in an 89% yield (see Table 1, entry 1). No desired product was detected when the reaction was conducted without an oxidant (Table 1, entry 2). Furthermore, nearly no product was afforded when the other oxidants were employed (Table 1, entries 3–5). Adjusting the amount of PhI(OAc)_2_ to 1.0 equiv. yielded the desired product **3aa** in a yield of 9% (Table 1, entry 6), while using 3.0 equiv. resulted in a 52% yield (Table 1, entry 7). We also evaluated the reaction temperature, finding that deviating from room temperature led to decreased yields (Table 1, entries 8–10). Additionally, we found that CH_3_CN was the optimal solvent compared to others (Table 1, entries 11–14). Finally, the yield of β-carbonyl sulfide **3aa** dropped to 75% when the reaction was performed in an air atmosphere (Table 1, entry 15).

**Figure 1:**
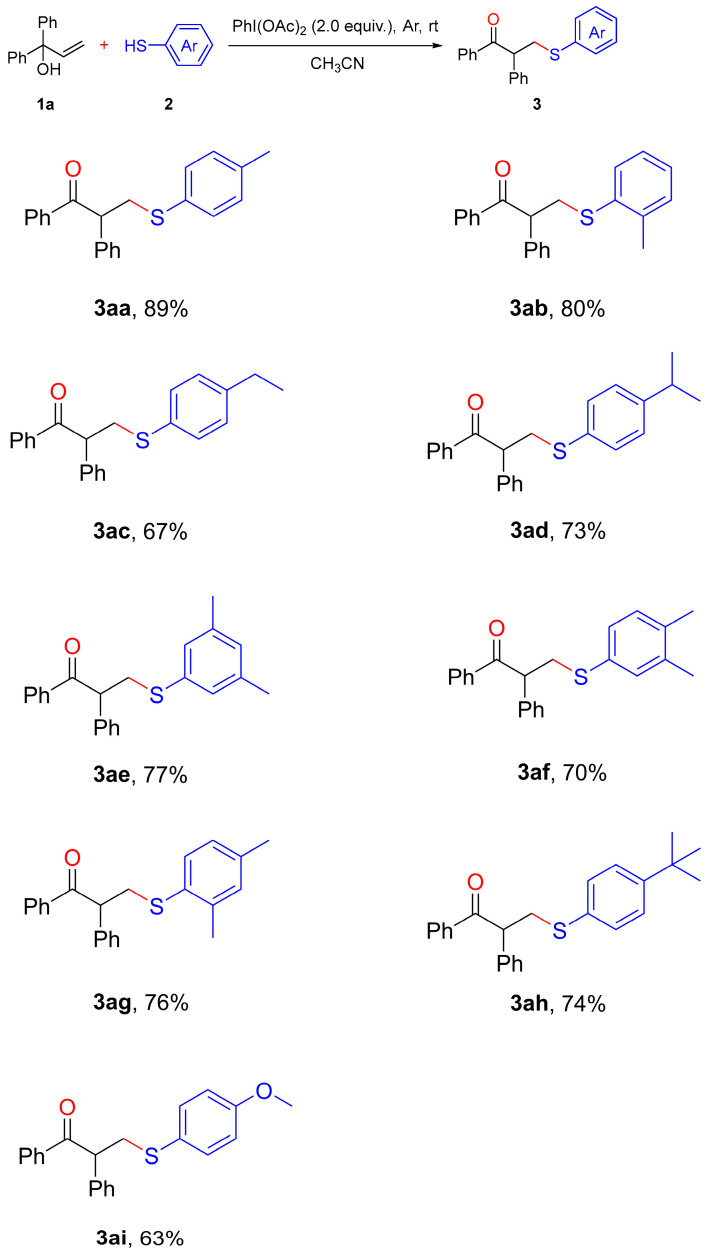
Substrate scope of the 1,2-transfer reaction of 1,1-diphenylprop-2-en-1-ol with various thiophenols ^a,b^. [a] Reaction conditions: **1a** (0.2 mmol); **2a** (0.4 mmol); PhI(OAc)_2_ (2 equiv.); CH_3_CN (2 mL); rt, 24 h. [b] Isolated yields.

With the above optimal conditions in hand, we investigated the scope for introducing thiophenols to the PhI(OAc)_2_-promoted 1,2-transfer reaction. As shown in Figure 1, all thiophenol derivatives, **2a**–**2j**, reacted smoothly with **1a** to obtain the β-carbonyl sulfides **3aa**–**3ai** in good to excellent yields. First, thiophenols bearing electron-neutral functional groups including methyl, ethyl, isopropyl, and *tert*-butyl were effectively reacted with 1,1-diphenylprop-2-en-1-ol, yielding corresponding β-carbonyl sulfides in 67–89% yields (Figure 1, **3aa**–**3ah**). Moreover, methoxy-substituted thiophenol can also deliver this desired transformation, giving the corresponding β-carbonyl sulfide in a yield of 73% (**3ai**).

**Figure 2:**
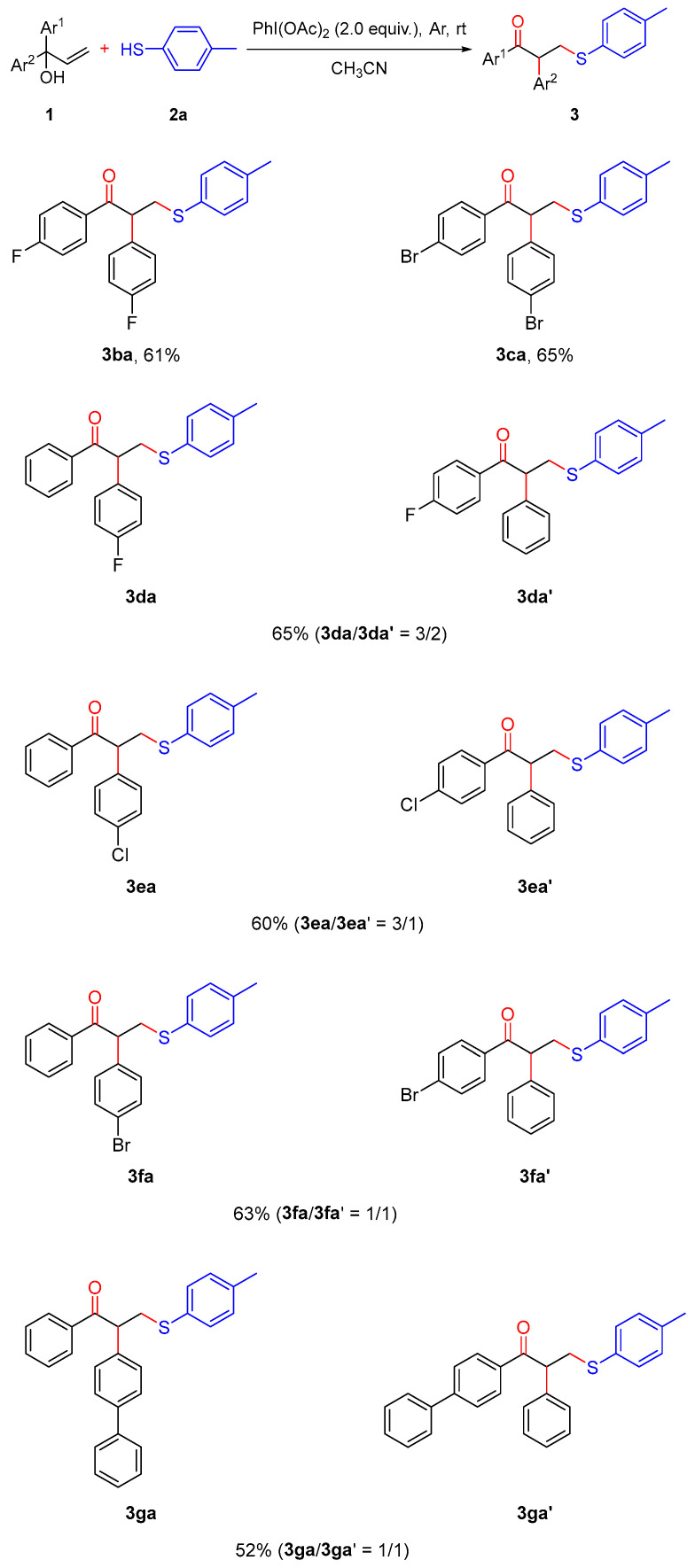
Scope of the 1,2-transfer reaction of various allyl alcohols with 4-methylthiophenol ^a,b^. [a] Reaction conditions: **1a** (0.2 mmol); **2a** (0.4 mmol); PhI(OAc)_2_ (2 equiv.); CH_3_CN (2 mL); rt, 24 h. [b] Isolated yields.

Next, a range of allyl alcohols were capable of participating in the 1,2-transfer reaction with high yields (**3ba**–**3ga**, 52–65%) in Figure 2. First, a series of symmetrical diaryl allylic alcohols were investigated. Delightfully, halogen-substituted allylic alcohols were successfully converted into the corresponding β-carbonyl sulfides **3ba**–**3ca**. In addition, allylic alcohols with two varying types of substituents on the phenyl ring were all compatible reaction partners (Figure 2, **3da**–**3ga** and **3da’**–**3ga’**). A variety of allylic alcohols with different electron-withdrawing or electron-donating substituents reacted nicely with 4-methylthiophenol in good yields to obtain β-carbonyl sulfides **3da**–**3ga** and their isomers **3da’**–**3ga’**.

**Scheme 2:**
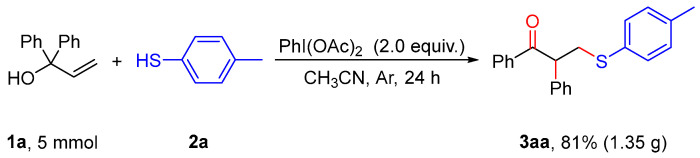
Gram-scale synthesis.

Moreover, the applicability of the PhI(OAc)_2_-promoted 1,2-transfer reaction was further demonstrated by a gram-scale reaction (Scheme 2). When the reaction was conducted with a metal-free approach, the expected β-carbonyl sulfide **3aa** could be obtained in a high isolated yield (81%).

**Scheme 3:**
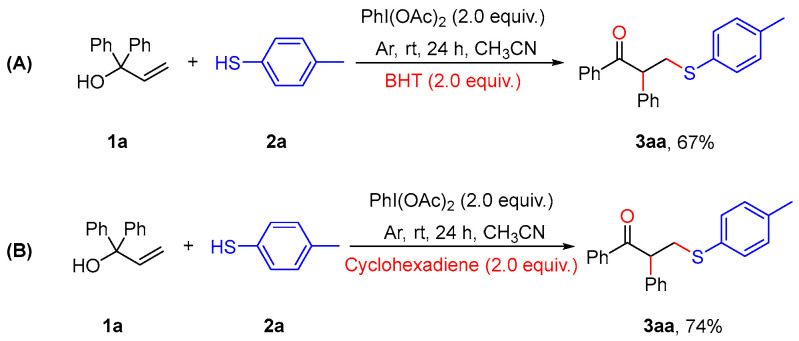
Control experiments.

To shed light on the mechanism of the 1,2-transfer reaction between 1,1-disubstituted allylic alcohols and thiophenols, a variety of control experiments were performed (Scheme 3). Running the model reaction in the presence of 2.0 equiv. of 2,6-di-tert-butyl-4-methylphenol (BHT) or 1,4-cyclohexadiene did not have much effect on the reaction, and β-carbonyl sulfide **3aa** was still the major product. These results indicated that the reaction involves the cationic mechanism pathway rather than the free radical process.

**Scheme 4:**
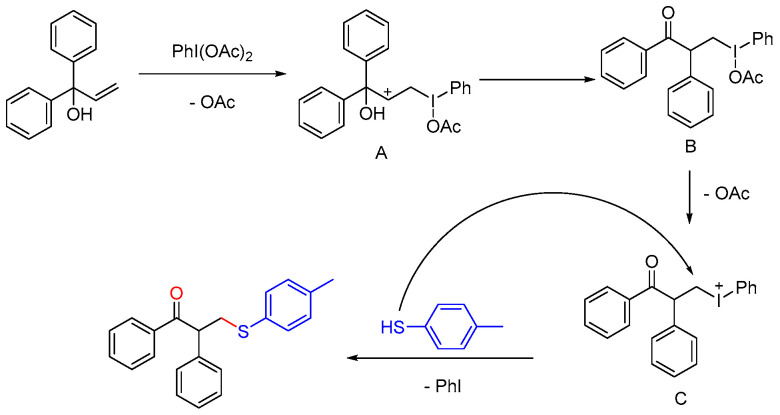
Proposed mechanism.

Based on the above experimental results and literature reports [41,42], a tentative mechanism for the PhI(OAc)_2_-promoted 1,2-transfer reaction is illustrated in Scheme 4. In the first place, PhI(OAc)_2_ attacks the 1,1-diaryl allyl alcohol double bond to form cation A. Then, the aryl group migrates to form intermediate B, and the acetate ion leaves to form iodide cation C. Finally, thiophenol attacks intermediate C and leaves a molecule of PhI to obtain the target product.

## 3. Materials and Methods

### 3.1. General Information

All materials, reagents, and solvents were purchased from commercial suppliers and were used without further purification. Analytical TLC was performed with silica gel GF254 plates, and the products were visualized by UV detection. Flash chromatography was carried out using silica gel 200–300. The ^1^H NMR (400 or 600 MHz) and ^13^C NMR (151 MHz) spectra were measured using CDCl_3_ as solvent (Supplementary Materials Figures S1–S30). All chemical shifts (δ) are reported in ppm and coupling constants (*J*) in Hz. High-resolution mass spectra (HR-MS) were recorded under electrospray ionization (ESI) conditions.

### 3.2. General Procedure for the 1,2-Transfer Reaction of 1,1-Diphenylprop-2-en-1-ol with Various Thiophenols

To a dried reaction tube (10 mL) with a magnetic bar, PhI(OAc)_2_ (0.4 mmol) was added, and the tube was then sealed. The tube was then charged with nitrogen. Then, α,α-diphenyl allylic alcohols (**1a**, 0.2 mmol), thiophenols (**2**, 0.4 mmol), and CH_3_CN (2 mL) were injected into the tube using a syringe. The reaction was performed at room temperature for 24 h. The reaction’s progress was monitored by TLC. After the reactions were completed, the reaction mixture was diluted with water (10 mL) and washed with EA (3 × 10 mL). The combined organic layers were dried over anhydrous Na_2_SO_4_ and concentrated under reduced pressure. The products were isolated by silica gel column chromatography (ethyl acetate/petroleum ether = 1:50 to 1:200).

### 3.3. General Procedure for the 1,2-Transfer Reaction of Various Allyl Alcohols with 4-Methylthiophenol

To a dried reaction tube (10 mL) with a magnetic bar, PhI(OAc)_2_ (0.4 mmol) was added, and the tube was then sealed. The tube was then charged with nitrogen. Then, α,α-diaryl allylic alcohols (**1**, 0.2 mmol), 4-methylthiophenol (**2a**, 0.4 mmol), and CH_3_CN (2 mL) were injected into the tube using a syringe. The reaction was performed at room temperature for 24 h. The reaction’s progress was monitored by TLC. After the reactions were completed, the reaction mixture was diluted with water (10 mL) and washed with EA (3 × 10 mL). The combined organic layers were dried over anhydrous Na_2_SO_4_ and concentrated under reduced pressure. The products were isolated by silica gel column chromatography (ethyl acetate/petroleum ether = 1:100 to 1:200).

### 3.4. Scale-Up Experiment

To a dried reaction tube (10 mL) with a magnetic bar PhI(OAc)_2_ (10 mmol) was added, and the tube was then sealed. The tube was then charged with nitrogen. Then, α,α-diaryl allylic alcohols (**1a**, 5 mmol), 4-methylthiophenol (**2a**, 10 mmol), and CH_3_CN (25 mL) were injected into the tube using a syringe. The reaction was performed at room temperature for 24 h. The reaction’s progress was monitored by TLC. After the reactions were completed, the reaction mixture was diluted with water (20 mL) and washed with EA (3 × 20 mL). The combined organic layers were dried over anhydrous Na_2_SO_4_ and concentrated under reduce pressure. The product **3aa** was isolated by silica gel column chromatography (ethyl acetate/petroleum ether = 1:100).

## 4. Conclusions

In summary, we have described a PhI(OAc)_2_-promoted 1,2-transfer reaction between allylic alcohols and thiophenols in high yields. This reaction method represents a powerful tool for synthesizing β-carbonyl sulfides, providing both high yields and rapid reaction times. Additionally, a tentative mechanism has been proposed. Further studies of the detailed reaction mechanism and its application are underway in our laboratory.

## Figures and Tables

**Table 1:** Screening of reaction conditions ^a^.

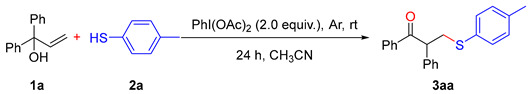		
Entry	Deviation from Standard Conditions	3aa Yield (%) ^b^
1	None	89
2	No oxidant	0
3	PhI(O_2_CCF_3_)_2_ as the oxidant	trace
4	K_2_S_2_O_8_ as the oxidant	0
5	DDQ as the oxidant	0
6	Using 1.0 equiv. of PhI(OAc)_2_	9
7	Using 3.0 equiv. of PhI(OAc)_2_	52
8	0 °C instead of rt	27
9	40 °C instead of rt	29
10	60 °C instead of rt	2
11	Toluene as the solvent	trace
12	DCE as the solvent	trace
13	Dioxane as the solvent	trace
14	DMF as the solvent	trace
15	Air instead of Ar	75

[a] Reaction conditions: **1a** (0.2 mmol); **2a** (0.4 mmol); PhI(OAc)_2_ (2 equiv.); CH_3_CN (2 mL); rt, 24 h. [b] Isolated yields.

## Data Availability

The data pertinent to this study are available in the article.

## Supplementary Materials

The following supporting information can be downloaded at: https://www.mdpi.com/article/10.3390/molecules29133112/s1. General experimental methods; Characterizations of compounds; NMR spectra of the products (Figures S1–S30).
