# Supplementary material for: PhI(OAc)2-Promoted 1,2-Transfer Reaction between 1,1-Disubstituted Allylic Alcohols and Thiophenols
Source: Molecules. 2024 Jun 29;29(13):3112. doi: 10.3390/molecules29133112 (PMC11243614; doi:10.3390/molecules29133112)
Supplement: Supplementary file 1 [file molecules-29-03112-s001.zip › molecules-3056771-supplementary.pdf]

Supporting Information

$PhI(OAc)_2$ Promoted 1,2-Transfer Reaction Between 1,1-Disubstituted Allylic Alcohols and Thiophenols

Guozhe GUO*, Wenduo LI, Jingjing ZHENG, Aping LIU, Qi ZHANG and Yatao WANG

Gansu Key Laboratory of Efficient Utilization of Oil and Gas Resources, College of Petroleum and Chemical Engineering, Longdong University, Qingyang, Gansu, 745000, China.

E-mail: guoguozhe2008@126.com.

Table of Contents

General experimental methods……………………………………………………………(Page S2)

Characterizations of compounds…………………………………………………………….(Page S2)

NMR spectra of the products…………………………………………………….……(Page S17)

Experimental Section

General Information

All materials, reagents and solvents were purchased from commercial suppliers and were used without further purification. The reactions were conducted under a nitrogen atmosphere. Analytical TLC was performed with silica gel GF254 plates, and the products were visualized by UV detection. Flash chromatography was carried out using silica gel 200−300. The ^{1}H NMR (400 or 600 MHz) and ^{13}C NMR (151 MHz) spectra were measured with $CDCl_3$ as solvent. All chemical shifts (δ) are reported in ppm and coupling constants (*J*) in Hz. High-resolution mass spectra (HR-MS) were recorded under electrospray ionization (ESI) conditions.

General procedure for sulfides of allylic alcohols

To a dried reaction tube (10 mL) with a magnetic bar was added $PhI(OAc)_2$ (0.4 mmol) and sealed. The tube was then charged with nitrogen. Then α,α-diaryl allylic alcohols (**1**, 0.2 mmol), thiophenols (**2**, 0.4 mmol) and CH_3CN (2 mL) were injected into the tube by syringe. The reaction mixture was performed at room temperature for 24 hours. The reaction progress was monitored by TLC. After the reactions were completed, the reaction mixture was diluted with water (10 mL) and washed with EA (3×10 mL). The combined organic layers dried over anhydrous Na_2SO_4 and concentrated under reduced pressure. The products were isolated by silica gel column chromatography (ethyl acetate/petroleum ether = 1:50 to 1:200).

Characterization of the products

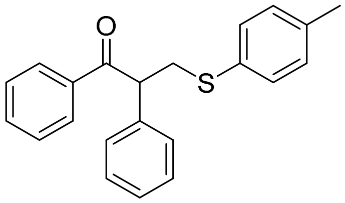

1,2-diphenyl-3-(p-tolylthio)propan-1-one (3aa) The desired pure product was obtained in 89% yield as a colorless liquid, R_f = 0.32 (20:1 petroleum ether/EtOAc). ^{1}H NMR (600 MHz, $CDCl_3$) δ 7.90 - 7.86 (m, 2H), 7.51 - 7.43 (m, 1H), 7.38 - 7.34 (m, 2H), 7.31 - 7.20 (m, 7H), 7.09 (d, *J* = 7.9 Hz, 2H), 4.78 (dd, *J* = 8.5, 5.8 Hz, 1H), 3.76 (dd, *J* = 13.3, 8.5 Hz, 1H), 3.26 (dd, *J* = 13.3, 5.8 Hz, 1H), 2.33 (s, 3H). ^{13}C NMR (151 MHz, $CDCl_3$) δ 198.3, 138.1, 136.5, 136.5, 133.0, 132.2, 130.6, 129.8, 129.1, 128.7, 128.5, 128.2, 127.6, 53.2, 38.1, 21.0. HRMS (ESI) exact mass calcd for $C_{22}H_{21}OS$ $[M+H]^+$ m/z 333.1313, found 333.1311.

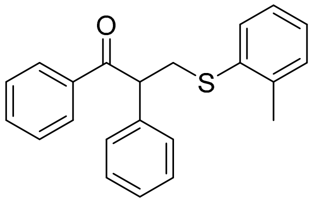

1,2-diphenyl-3-(o-tolylthio)propan-1-one (3ab) The desired pure product was obtained in 80% yield as a colorless liquid, R_f = 0.32 (20:1 petroleum ether/EtOAc). ^{1}H NMR (600 MHz, $CDCl_3$) δ 7.89 (m, 2H), 7.49 - 7.45 (m, 3H), 7.38 - 7.31 (m, 2H), 7.30 - 7.28 (m, 4H), 7.24 - 7.20 (m, 1H), 7.18 - 7.09 (m, 3H), 4.81 (dd, J = 8.6, 5.7 Hz, 1H), 3.80 (dd, J = 13.2, 8.6 Hz, 1H), 3.27 (dd, J = 13.2, 5.6 Hz, 1H), 2.28 (s, 3H). ^{13}C NMR (151 MHz, $CDCl_3$) δ 198.2, 138.3, 138.1, 136.4, 135.2, 133.1, 130.3, 129.1, 128.7, 128.7, 128.5, 128.1, 127.6, 126.4, 126.1, 53.1, 36.6, 20.4. HRMS (ESI) exact mass calcd for $C_{22}H_{21}OS$ $[M+H]^+$ m/z 333.1313, found 333.1315.

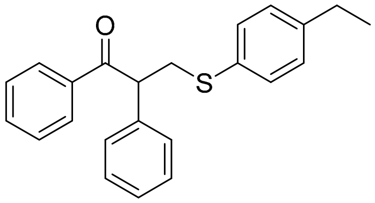

3-((4-ethylphenyl)thio)-1,2-diphenylpropan-1-one (3ac) The desired pure product was obtained in 67% yield as a colorless liquid(d/r = 9:5) , R_f = 0.32 (20:1 petroleum ether/EtOAc). ^{1}H NMR (400 MHz, $CDCl_3$) δ 8.05 - 7.76 (m, 2H), 7.49 - 7.45 (m, 1H), 7.39 - 7.34 (m, 3H), 7.32 - 7.25 (m, 6H), 7.18 (d, J = 3.8 Hz, 1H), 7.13 (d, J = 8.5 Hz, 1H), 4.92 - 4.72 (m, 1H), 3.89 - 3.73 (m, 1H), 3.33 - 3.21 (m, 1H), 2.70 - 2.58 (m, 2H), 1.24 (t, J = 7.6 Hz, 1.8H), 1.13 (t, J = 7.5 Hz, 1.1H). ^{13}C NMR (151 MHz, $CDCl_3$) δ 198.3, 198.2, 144.3, 142.9, 138.1, 138.1, 136.5, 136.5, 134.6, 133.1, 133.1, 132.1, 132.4, 130.6, 129.1, 129.1, 128.7, 128.7, 128.6, 128.6, 128.5, 128.5, 128.2, 128.1, 127.6, 127.6, 126.5, 126.4, 53.3, 53.1, 38.1, 37.1, 28.4, 26.9, 15.5, 14.7. HRMS (ESI) exact mass calcd for $C_{23}H_{23}OS$ $[M+H]^+$ m/z 347.1470, found 347.1472.

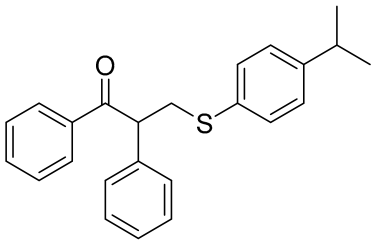

3-((4-isopropylphenyl)thio)-1,2-diphenylpropan-1-one (3ad) The desired pure product was obtained in 73% yield as a colorless liquid, R_f = 0.32 (20:1 petroleum ether/EtOAc). ^{1}H NMR (600 MHz, $CDCl_3$) δ 7.88 (dd, J = 8.4, 1.2 Hz, 2H), 7.50 - 7.43 (m, 1H), 7.38 - 7.34 (m, 2H), 7.31 - 7.26 (m, 6H), 7.24 - 7.20 (m, 1H), 7.16 - 7.12 (m, 2H), 4.80 (dd, J = 8.4, 5.9 Hz, 1H), 3.77 (dd, J = 13.3, 8.4 Hz, 1H), 3.27 (dd, J

= 13.3, 5.9 Hz, 1H), 2.92 - 2.84 (m, 1H), 1.24 (d, J = 6.9 Hz, 6H). ^{13}C NMR (151 MHz, $CDCl_3$) δ 198.3, 147.5, 138.1, 136.5, 133.0, 132.6, 130.5, 129.1, 128.7, 128.5, 128.2, 127.6, 127.1, 53.3, 38.0, 33.7, 23.9. HRMS (ESI) exact mass calcd for $C_{24}H_{25}OS$ $[M+H]^+$ m/z 361.1626, found 361.1630.

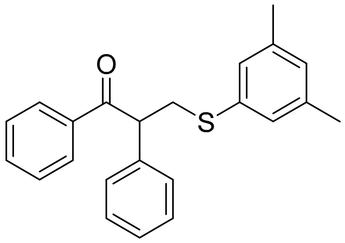

3-((3,5-dimethylphenyl)thio)-1,2-diphenylpropan-1-one (3ae) The desired pure product was obtained in 77% yield as a colorless liquid, R_f = 0.32 (20:1 petroleum ether/EtOAc). ^{1}H NMR (400 MHz, $CDCl_3$) δ 7.91 (t, J = 7.8 Hz, 2H), 7.49 (dd, J = 8.6, 6.1 Hz, 1H), 7.37 (t, J = 7.8 Hz, 3H), 7.31 (dd, J = 9.1, 4.1 Hz, 4H), 7.09 (s, 1H), 6.94 (s, 1H), 6.55 (s, 1H), 4.83 (ddd, J = 17.6, 8.3, 5.9 Hz, 1H), 3.81 (ddd, J = 21.3, 13.1, 8.4 Hz, 1H), 3.33 (ddd, J = 27.9, 13.1, 5.8 Hz, 1H), 2.37 (s, 2H), 2.26 (s, 3H), 2.20 (s, 3H). ^{13}C NMR (151 MHz, $CDCl_3$) δ 198.1, 144.4, 138.6, 137.9, 137.4, 137.3, 136.3, 133.2, 129.2, 128.8, 128.6, 128.3, 128.1, 127.7, 126.8, 123.2, 53.3, 36.6, 21.9, 21.3. HRMS (ESI) exact mass calcd for $C_{23}H_{23}OS$ $[M+H]^+$ m/z 347.1470, found 347.1475.

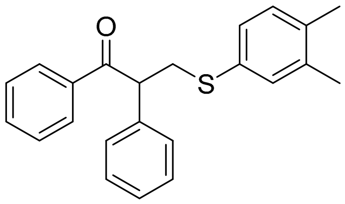

3-((3,4-dimethylphenyl)thio)-1,2-diphenylpropan-1-one (3af) The desired pure product was obtained in 70% yield as a colorless liquid, R_f = 0.32 (20:1 petroleum ether/EtOAc). ^{1}H NMR (600 MHz, $CDCl_3$) δ 7.90 - 7.86 (m, 2H), 7.47 (m, 1H), 7.38 - 7.34 (m, 2H), 7.31 - 7.26 (m, 4H), 7.24 - 7.19 (m, 1H), 7.12 - 7.08 (m, 2H), 7.05 (d, J = 7.5 Hz, 1H), 4.79 (dd, J = 8.5, 5.8 Hz, 1H), 3.76 (dd, J = 13.3, 8.5 Hz, 1H), 3.25 (dd, J = 13.3, 5.8 Hz, 1H), 2.23 (s, 3H), 2.20 (s, 3H). ^{13}C NMR (151 MHz, $CDCl_3$) δ 198.4, 138.1, 137.4, 136.1, 135.2, 133.0, 132.3, 131.9, 130.2, 129.0, 128.7, 128.5, 128.2, 128.0, 127.5, 53.2, 38.1, 19.7, 19.3. HRMS (ESI) exact mass calcd for $C_{23}H_{23}OS$ $[M+H]^+$ m/z 347.1470, found 347.1475.

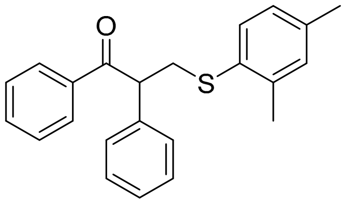

3-((2,4-dimethylphenyl)thio)-1,2-diphenylpropan-1-one (3ag) The desired pure product was obtained in 76% yield as a colorless liquid, R_f = 0.32 (20:1 petroleum ether/EtOAc). ^{1}H NMR (400 MHz, $CDCl_3$)

δ 7.90 (d, *J* = 7.4 Hz, 2H), 7.49 (t, *J* = 7.3 Hz, 1H), 7.37 (t, *J* = 7.6 Hz, 2H), 7.32 - 7.27 (m, 4H), 7.25 (dd, *J* = 6.8, 3.5 Hz, 2H), 7.04 - 6.90 (m, 2H), 4.80 (dd, *J* = 8.6, 5.6 Hz, 1H), 3.76 (dd, *J* = 13.1, 8.7 Hz, 1H), 3.21 (dd, *J* = 13.1, 5.5 Hz, 1H), 2.31 (s, 3H), 2.28 (s, 3H). ^{13}C NMR (151 MHz, $CDCl_3$) δ 198.3, 138.9, 138.2, 136.5, 136.4, 133.0, 131.3, 131.2, 130.2, 129.1, 128.7, 128.5, 128.1, 127.6, 127.2, 53.1, 37.2, 20.9, 20.4. HRMS (ESI) exact mass calcd for $C_{23}H_{23}OS$ $[M+H]^+$ m/z 347.1470, found 347.1473.

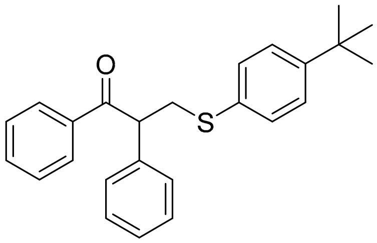

3-((4-(tert-butyl)phenyl)thio)-1,2-diphenylpropan-1-one (3ah) The desired pure product was obtained in 74% yield as a colorless liquid, R_f = 0.32 (20:1 petroleum ether/EtOAc). ^{1}H NMR (400 MHz, $CDCl_3$) δ 7.91 (d, *J* = 7.9 Hz, 2H), 7.48 (t, *J* = 7.2 Hz, 1H), 7.37 (t, *J* = 7.6 Hz, 2H), 7.31 (d, *J* = 3.4 Hz, 8H), 7.27 - 7.20 (m, 1H), 4.84 (dd, *J* = 8.1, 6.0 Hz, 1H), 3.80 (dd, *J* = 13.2, 8.4 Hz, 1H), 3.30 (dd, *J* = 13.2, 5.8 Hz, 1H), 1.33 (s, 9H). ^{13}C NMR (151 MHz, $CDCl_3$) δ 198.3, 149.7, 138.1, 136.5, 133.0, 132.4, 130.0, 129.1, 128.7, 128.5, 128.2, 127.6, 126.0, 53.4, 37.9, 34.5, 31.3. HRMS (ESI) exact mass calcd for $C_{25}H_{27}OS$ $[M+H]^+$ m/z 375.1783, found 375.1782.

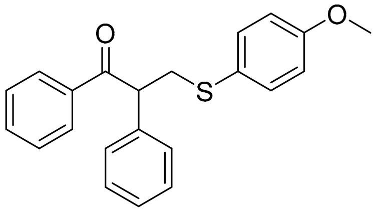

3-((4-methoxyphenyl)thio)-1,2-diphenylpropan-1-one (3ai) The desired pure product was obtained in 63% yield as a colorless liquid, R_f = 0.35 (5:1 petroleum ether/EtOAc). ^{1}H NMR (400 MHz, $CDCl_3$) δ 7.93 - 7.86 (m, 2H), 7.52 - 7.46 (m, 1H), 7.43 - 7.34 (m, 2H), 7.34 - 7.30 (m, 2H), 7.30 - 7.16 (m, 5H), 6.90 - 6.76 (m, 2H), 4.76 (dd, *J* = 8.6, 5.7 Hz, 1H), 3.81 (s, 3H), 3.72 (dd, *J* = 13.3, 8.7 Hz, 1H), 3.19 (dd, *J* = 13.3, 5.7 Hz, 1H). ^{13}C NMR (151 MHz, $CDCl_3$) δ 198.3, 159.1, 138.1, 136.5, 133.7, 133.1, 129.1, 128.7, 128.5, 128.2, 127.6, 126.1, 114.7, 55.3, 53.2, 39.5. HRMS (ESI) exact mass calcd for $C_{22}H_{21}O_2S$ $[M+H]^+$ m/z 349.1262, found 349.1266.

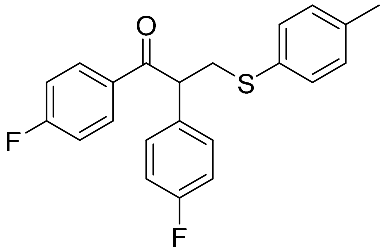

1,2-bis(4-fluorophenyl)-3-(p-tolylthio)propan-1-one (3ba) The desired pure product was obtained in 61% yield as a colorless liquid, R_f = 0.33 (10:1 petroleum ether/EtOAc). ^{1}H NMR (400 MHz, $CDCl_3$) δ 7.94 - 7.84 (m, 2H), 7.29 - 7.18 (m, 4H), 7.10 (d, *J* = 7.9 Hz, 2H), 7.04 (t, *J* = 12.1, 5.1 Hz, 2H), 6.98 (t, *J* = 8.6 Hz, 2H), 4.71 (dd, *J* = 8.2, 6.2 Hz, 1H), 3.71 (dd, *J* = 13.4, 8.2 Hz, 1H), 3.22 (dd, *J* = 13.4, 6.1 Hz, 1H), 2.34 (s, 3H). ^{13}C NMR (151 MHz, $CDCl_3$) δ 196.8, 165.7(d, *J* = 255.7Hz), 162.2 (d, *J* = 246.9 Hz), 136.8, 133.6 (d, *J* = 3.3 Hz), 132.7 (d, *J* = 3.1 Hz), 131.8, 131.3(d, *J* = 9.4 Hz), 130.7, 129.8, 129.7 (d, *J* = 8.1 Hz), 116.1 (d, *J* = 21.6 Hz), 115.7 (d, *J* = 21.9 Hz), 52.3, 38.2, 21.0. HRMS (ESI) exact mass calcd for $C_{22}H_{19}F_2OS$ $[M+H]^+$ m/z 369.1125, found 369.1122.

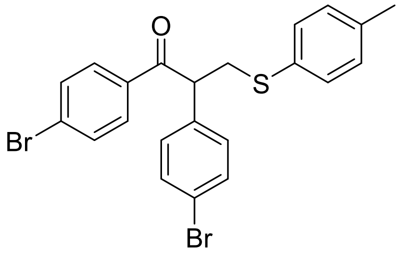

1,2-bis(4-bromophenyl)-3-(p-tolylthio)propan-1-one (3ca) The desired pure product was obtained in 65% yield as a colorless liquid, R_f = 0.33 (10:1 petroleum ether/EtOAc). ^{1}H NMR (400 MHz, $CDCl_3$) δ 7.74 - 7.62 (m, 4H), 7.50 (d, *J* = 8.5 Hz, 2H), 7.40 (d, *J* = 8.4 Hz, 2H), 7.22 (d, *J* = 8.1 Hz, 2H), 7.13 - 7.07 (m, 4H), 4.65 (dd, *J* = 8.0, 6.2 Hz, 1H), 3.69 (dd, *J* = 13.4, 8.1 Hz, 1H), 3.21 (dd, *J* = 13.4, 6.2 Hz, 1H), 2.33 (s, 3H). ^{13}C NMR (151 MHz, $CDCl_3$) δ 197.0, 136.9, 136.6, 134.9, 132.3, 131.9, 131.7, 131.6, 131.4, 130.8, 130.1, 129.9, 129.8, 128.6, 121.9, 52.6, 37.9, 21.1. HRMS (ESI) exact mass calcd for $C_{22}H_{19}Br_2OS$ $[M+H]^+$ m/z 490.9503, found 490.9500.

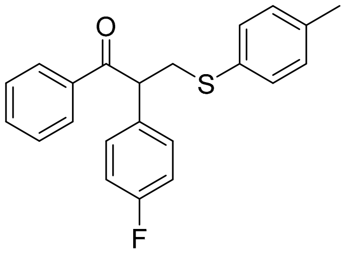

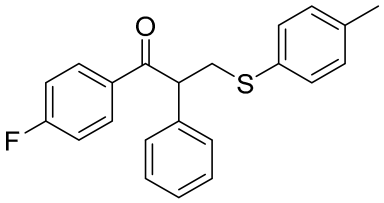

2-(4-fluorophenyl)-1-phenyl-3-(p-tolylthio)propan-1-one (3da) and 1-(4-fluorophenyl)-2-phenyl-3-(p-tolylthio)propan-1-one (3da') The desired pure product was obtained in 65% yield as a colorless liquid (**3da**/**3da'** = 3/2), R_f = 0.33 (10:1 petroleum ether/EtOAc). ^{1}H NMR (400 MHz, $CDCl_3$) δ 7.99 - 7.83 (m, 2H), 7.50 (t, *J* = 7.4 Hz, 0.5H), 7.38 (t, *J* = 7.7 Hz, 0.75H), 7.33 - 7.21 (m, 6H), 7.11 (d, *J* = 7.7 Hz, 2H), 7.03 (t, *J* = 8.6 Hz, 1.2H), 6.98 (t, *J* = 8.7 Hz, 0.8H), 4.77 (dd, *J* = 8.0, 6.5 Hz,0.4H), 4.73 (dd, *J* = 8.6, 5.7 Hz, 0.6H), 3.81 - 3.68 (m, 1H), 3.25 (ddd, *J* = 13.3, 5.9, 1.9 Hz, 1H), 2.34 (s, 3H). ^{13}C NMR (151 MHz, $CDCl_3$) δ 198.3, 196.7, 166.6 (d, *J* = 255.3 Hz), 162.2 (d, 246.6 Hz), 137.9, 136.7, 136.6, 133.2, 132.1, 131.4 (d, *J* = 9.4 Hz), 130.7, 130.6, 129.8, 129.8, 129.77, 129.1, 128.6 (d, *J* = 21.5 Hz), 128.3,

128.1, 127.7, 116.0 (d, J = 21.5 Hz), 115.6 (d, J = 21.9 Hz), 53.3, 52.25, 38.2, 38.1, 21.0. HRMS (ESI) exact mass calcd for $C_{22}H_{20}FOS$ $[M+H]^+$ m/z 351.1219, found 351.1217.

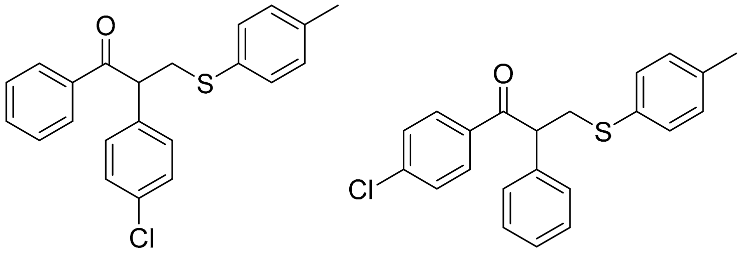

1-(4-chlorophenyl)-2-phenyl-3-(p-tolylthio)propan-1-one (3ea) and 2-(4-chlorophenyl)-1-phenyl-3-(p-tolylthio)propan-1-one (3ea') The desired pure product was obtained in 60% yield as a colorless liquid (**3ea/3ea'** = 3/1), R_f = 0.33 (10:1 petroleum ether/EtOAc). ^{1}H NMR (400 MHz, $CDCl_3$) δ 7.85 - 7.74 (m, 3H), 7.53 - 7.44 (m, 1H), 7.36 - 7.27 (m, 4H), 7.27 - 7.21 (m, 3H), 7.10 (d, J = 8.4 Hz, 2H), 4.71 (dd, J = 8.6, 5.6 Hz, 1H), 3.75 (dd, J = 13.3, 8.7 Hz, 1H), 3.24 (dd, J = 13.3, 5.6 Hz, 1H), 2.34 (s, 3H). ^{13}C NMR (151 MHz, $CDCl_3$) δ 197.1, 139.5, 136.7, 131.4, 130.6, 130.1, 129.9, 129.8, 129.2, 128.8, 128.6, 128.4, 128.1, 127.7, 53.3, 38.0, 21.0. HRMS (ESI) exact mass calcd for $C_{22}H_{20}ClOS$ $[M+H]^+$ m/z 367.0923, found 367.0920.

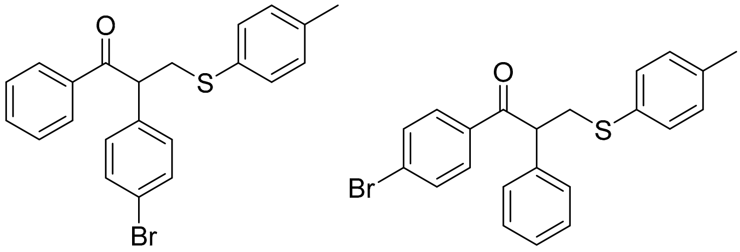

1-(4-bromophenyl)-2-phenyl-3-(p-tolylthio)propan-1-one (3fa) and 2-(4-bromophenyl)-1-phenyl-3-(p-tolylthio)propan-1-one (3fa') The desired pure product was obtained in 63% yield as a colorless liquid (**3fa/3fa'** = 1/1), R_f = 0.33 (10:1 petroleum ether/EtOAc). ^{1}H NMR (400 MHz, $CDCl_3$) δ 7.87 - 7.83 (m, 1H), 7.75 - 7.71 (m, 1H), 7.52 - 7.47 (m, 1H), 7.43 - 7.37 (m, 2H), 7.29 (d, J = 6.9 Hz, 1H), 7.25 - 7.21 (m, 3H), 7.16 - 7.13 (m, 1H), 7.10 (dd, J = 8.1, 1.8 Hz, 2H), 4.76 - 4.67 (m, 1H), 3.73 (ddd, J = 21.4, 11.2, 7.1 Hz, 1H), 3.24 (dd, J = 13.4, 5.9 Hz, 1H), 2.34 (s, 3H). ^{13}C NMR (151 MHz, $CDCl_3$) δ 198.0, 197.3, 137.8, 137.0, 136.8, 136.7, 136.2, 135.2, 133.3, 132.2, 131.8, 130.8, 130.7, 130.2, 129.9, 129.8, 129.8, 129.2, 128.7, 128.6, 128.1, 127.8, 121.7, 53.3, 52.5, 38.1, 38.0, 21.1. HRMS (ESI) exact mass calcd for $C_{22}H_{20}BrOS$ $[M+H]^+$ m/z 411.0418, found 411.0422.

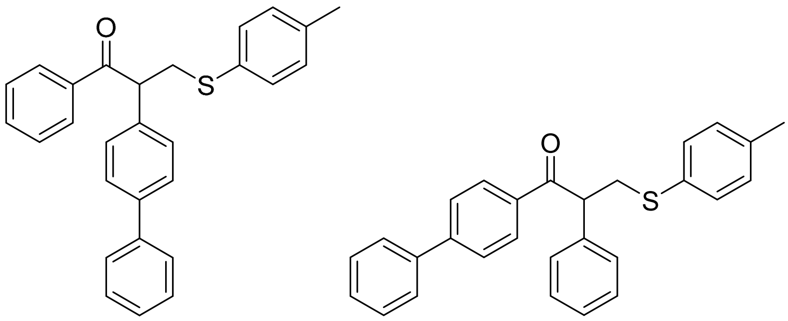

2-([1,1'-biphenyl]-4-yl)-1-phenyl-3-(p-tolylthio)propan-1-one (3ga) and 1-([1,1'-biphenyl]-4-yl)-2-phenyl-3-(p-tolylthio)propan-1-one (3ga') The desired pure product was obtained in 52% yield as a colorless liquid (**3ga**/**3ga'** = 1/1), R_f = 0.33 (10:1 petroleum ether/EtOAc). ^{1}H NMR (400 MHz, $CDCl_3$) δ 7.96 (dd, *J* = 17.5, 8.1 Hz, 2H), 7.64 - 7.49 (m, 5H), 7.47 - 7.23 (m, 9H), 7.12 (d, *J* = 6.8 Hz, 2H), 4.84 (dd, *J* = 14.0, 7.3 Hz, 1H), 3.81 (dd, *J* = 13.2, 8.5 Hz, 1H), 3.31 (td, *J* = 13.0, 5.8 Hz, 1H), 2.35 (s, 3H). ^{13}C NMR (151 MHz, $CDCl_3$) δ 198.3, 197.9, 145.7, 140.5, 140.4, 139.8, 138.2, 137.0, 136.6, 136.5, 136.5, 135.2, 133.1, 132.2, 132.2, 130.7, 130.6, 129.8, 129.3, 129.1, 128.9, 128.8, 128.7, 128.6, 128.5, 128.2, 128.18, 127.8, 127.6, 127.4, 127.2, 127.2, 127.0, 53.2, 52.9, 38.1, 38.1, 21.1. HRMS (ESI) exact mass calcd for $C_{28}H_{25}OS$ $[M+H]^+$ m/z 409.1626, found 409.1621.

NMR spectra of the products

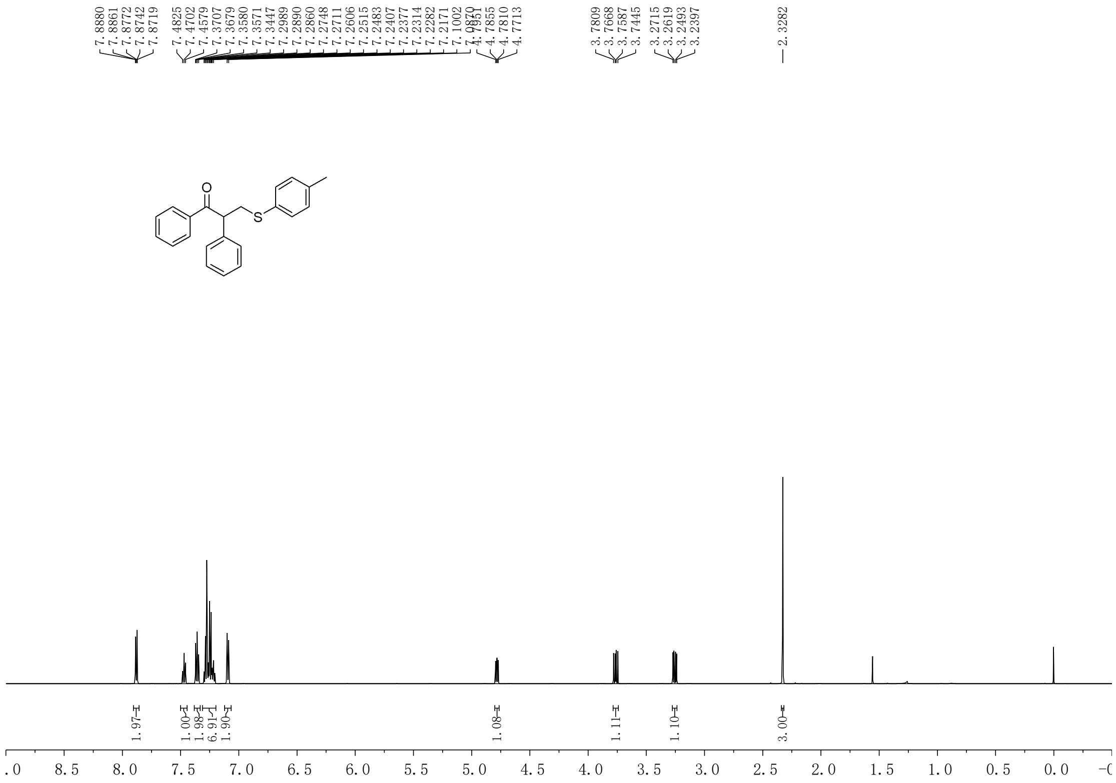

Figure S1: The ^{1}H NMR (600 MHz) of compound **3aa**

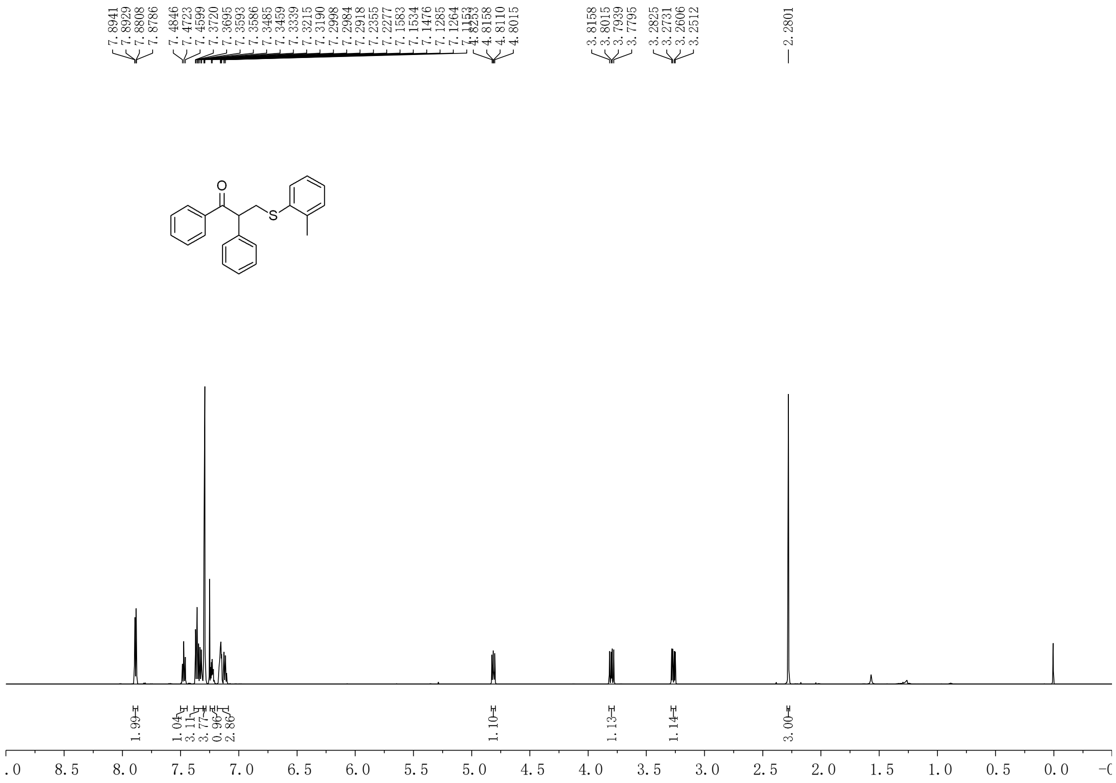

Figure S2: The ^{1}H NMR (600 MHz) of compound **3ab**

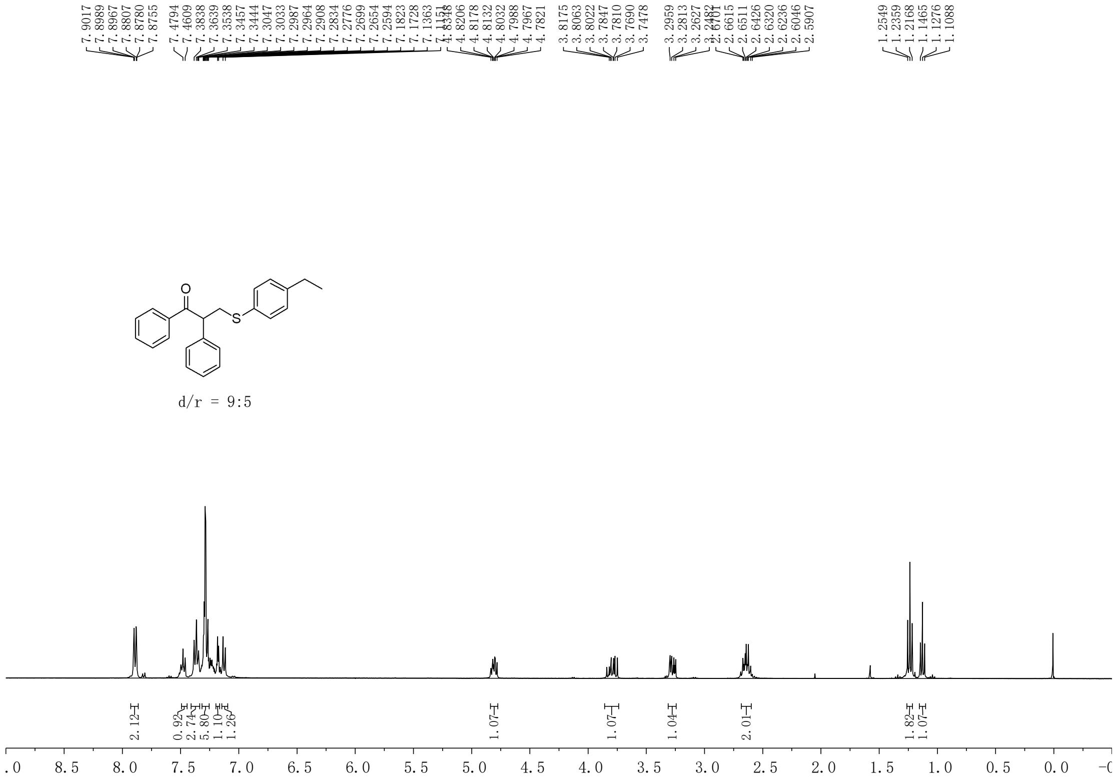

Figure S3: The ¹H NMR (400 MHz) of compound **3ac**

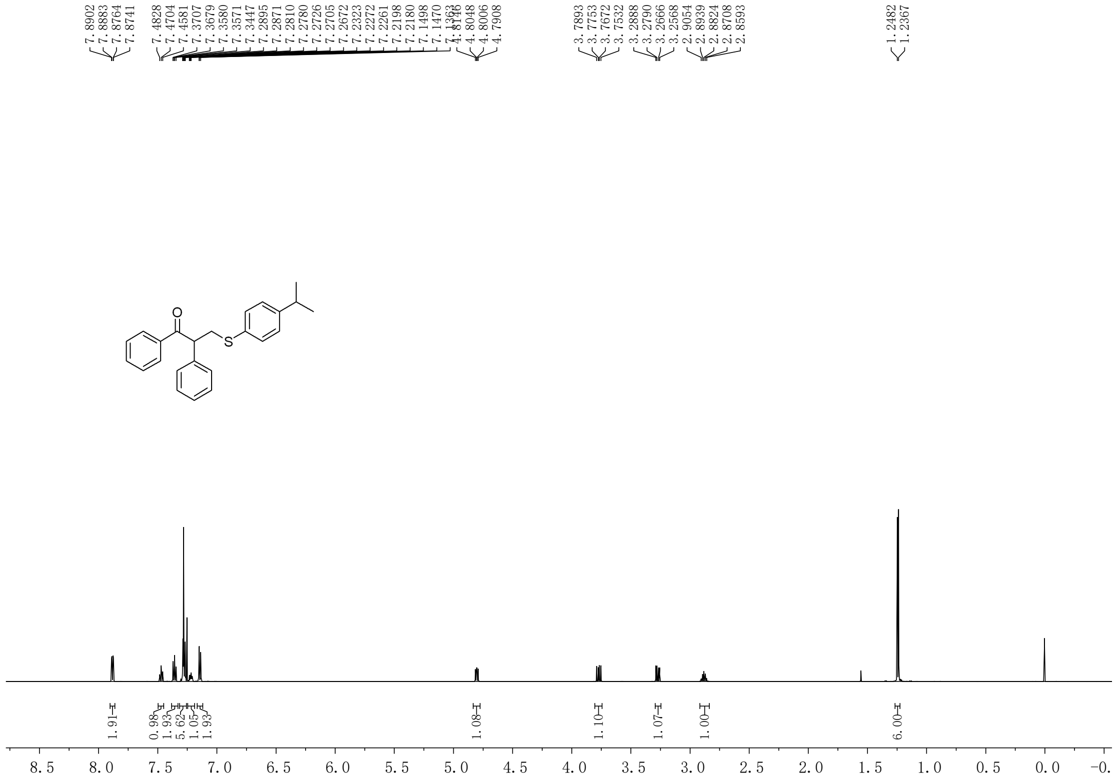

Figure S4: The ¹H NMR (600 MHz) of compound **3ad**

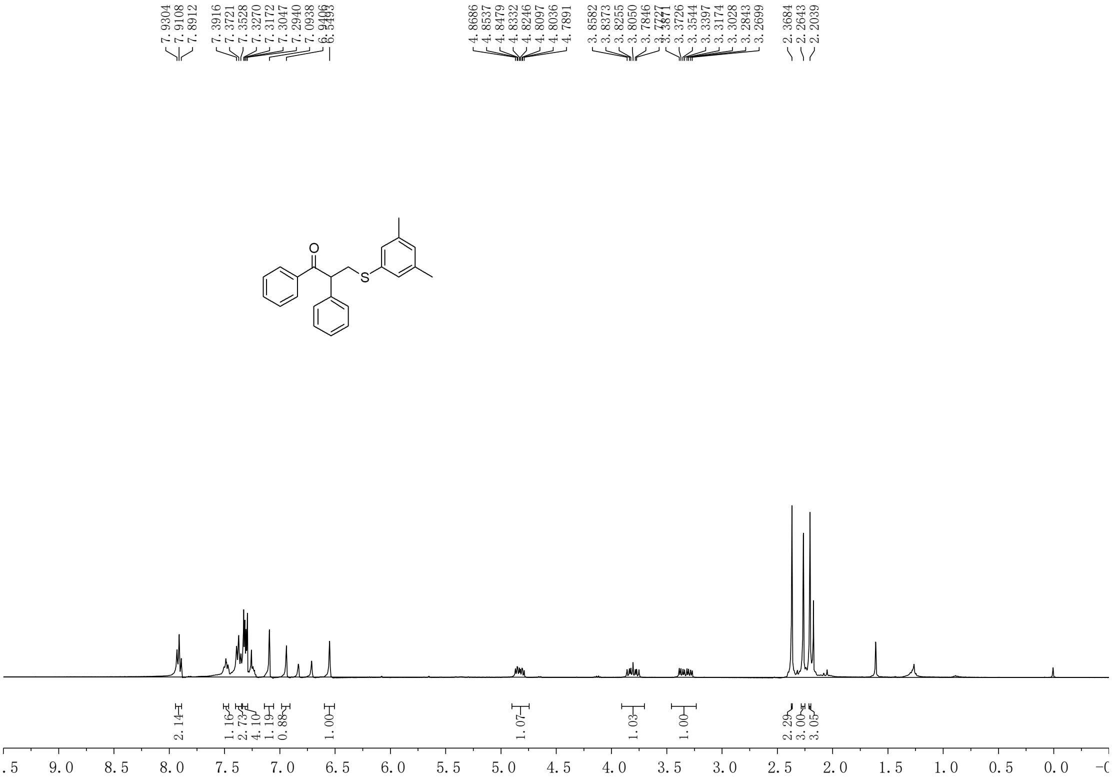

Figure S5: The ^{1}H NMR (400 MHz) of compound **3ae**

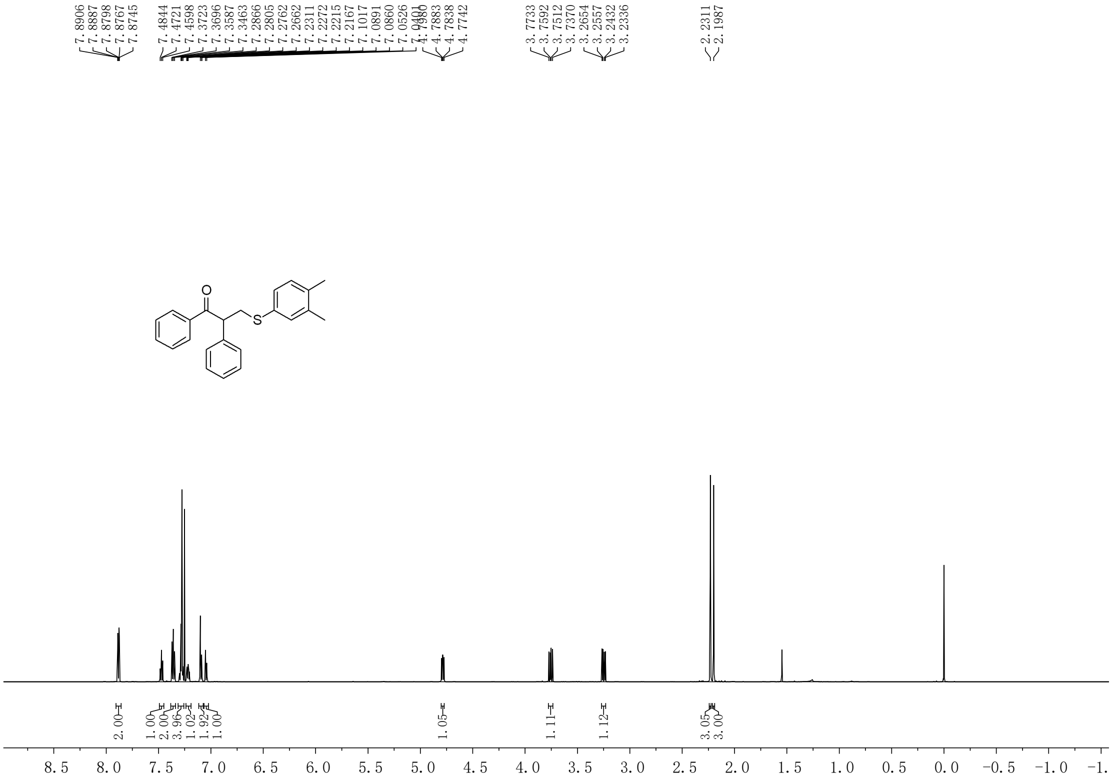

Figure S6: The ^{1}H NMR (600 MHz) of compound **3af**

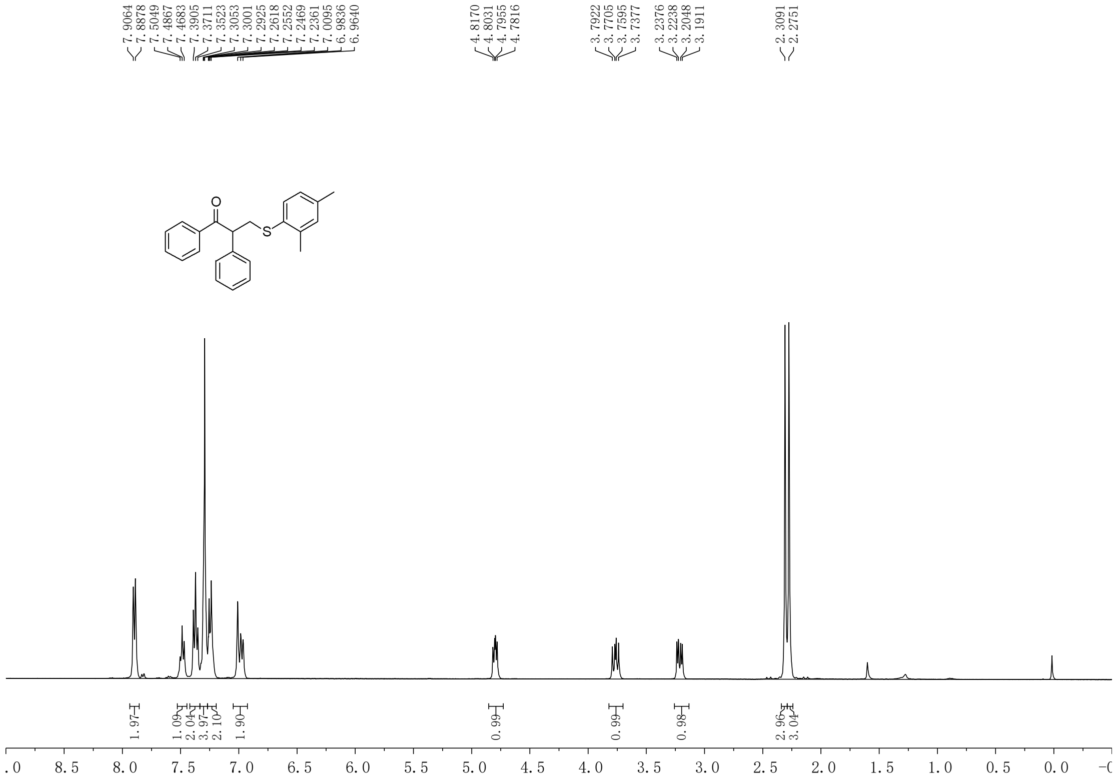

Figure S7: The ^{1}H NMR (400 MHz) of compound **3ag**

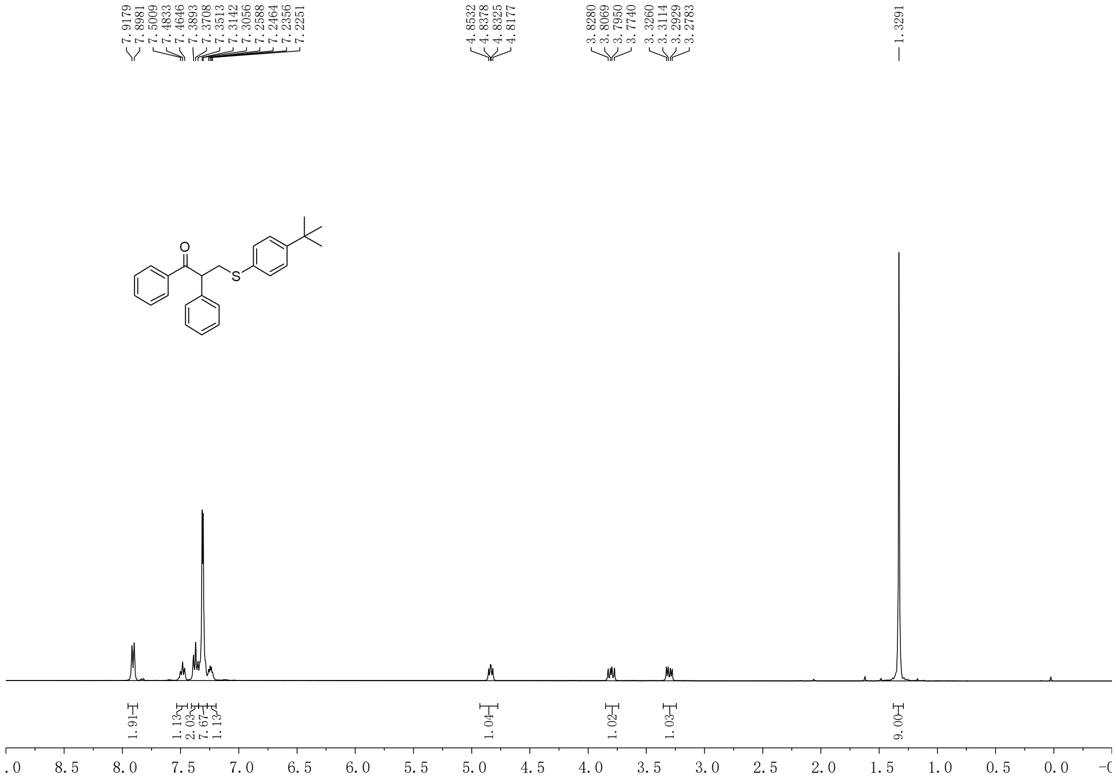

Figure S8: The ^{1}H NMR (400 MHz) of compound **3ah**

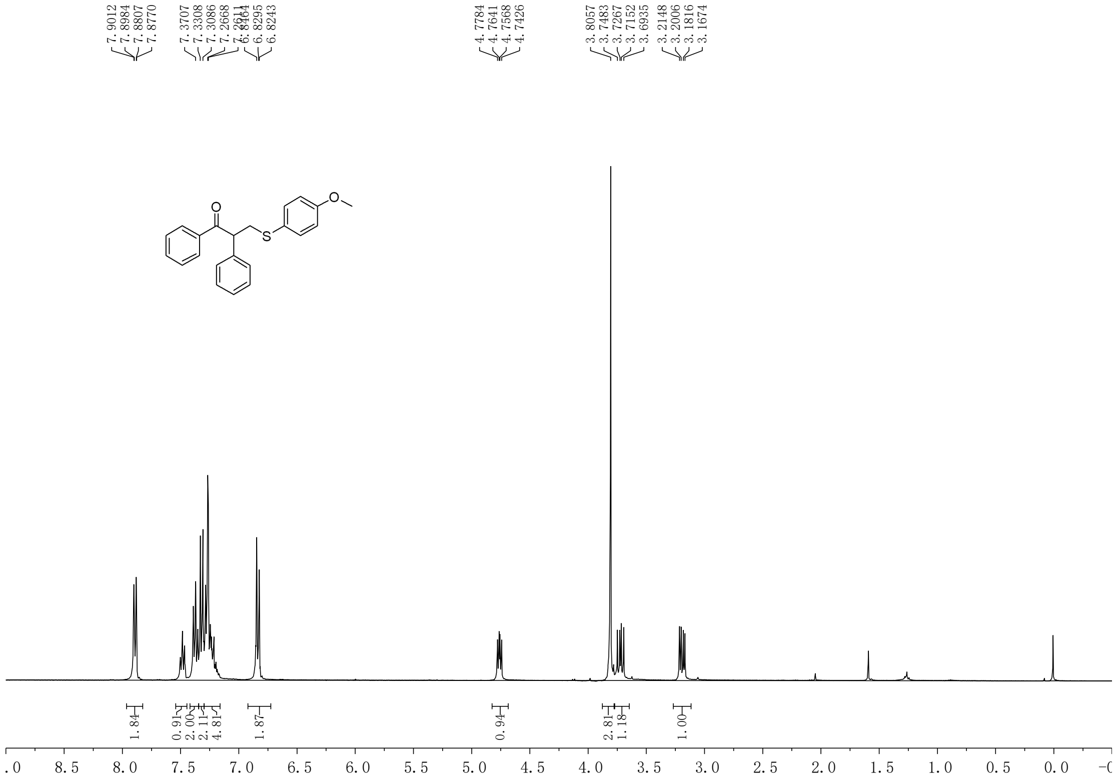

Figure S9: The ¹H NMR (400 MHz) of compound **3ai**

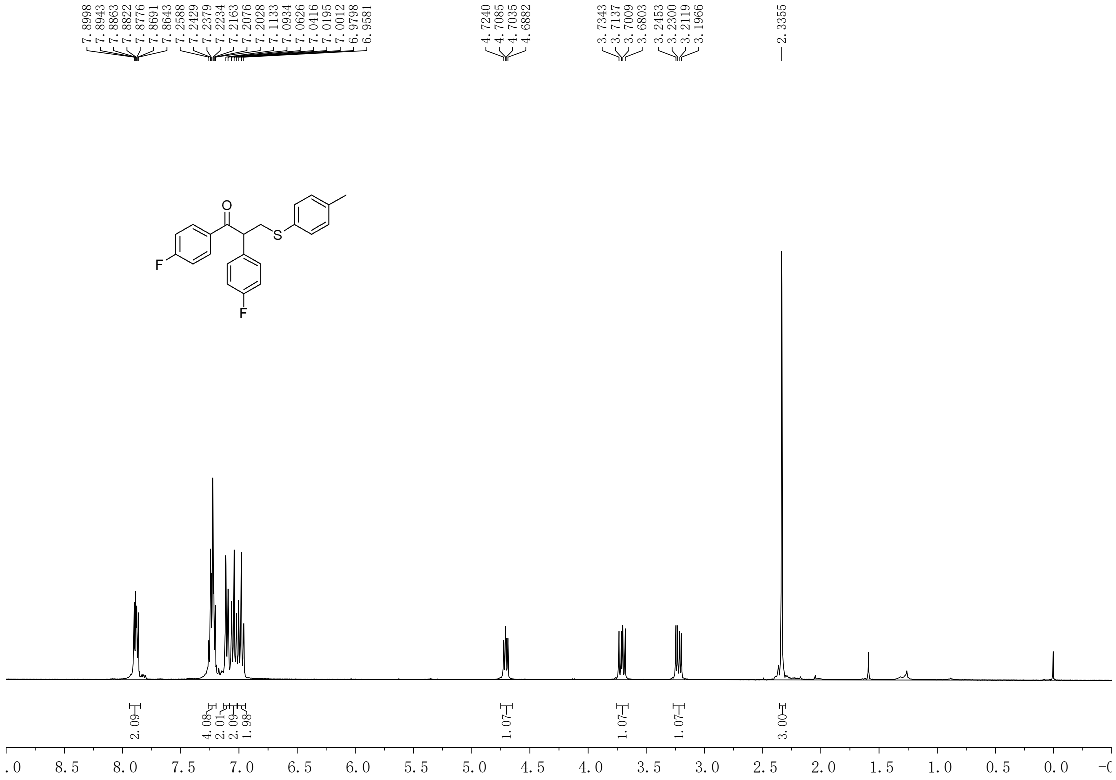

Figure S10: The ¹H NMR (400 MHz) of compound **3ba**

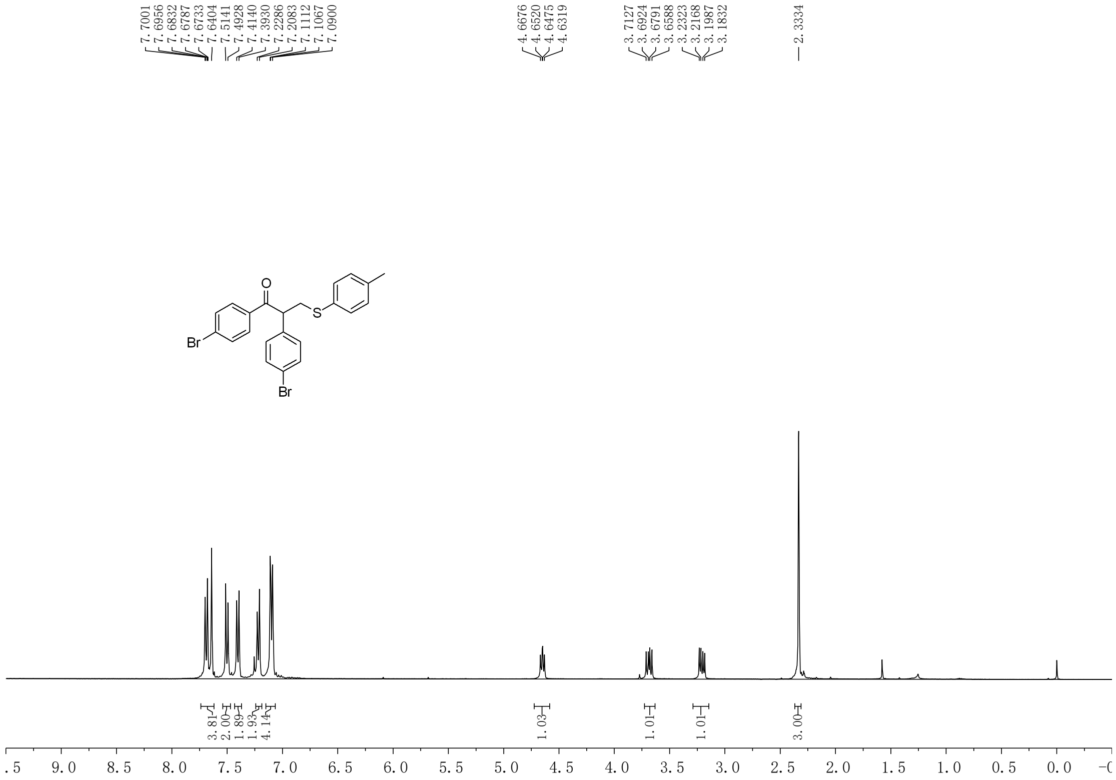

Figure S11: The ¹H NMR (400 MHz) of compound **3ca**

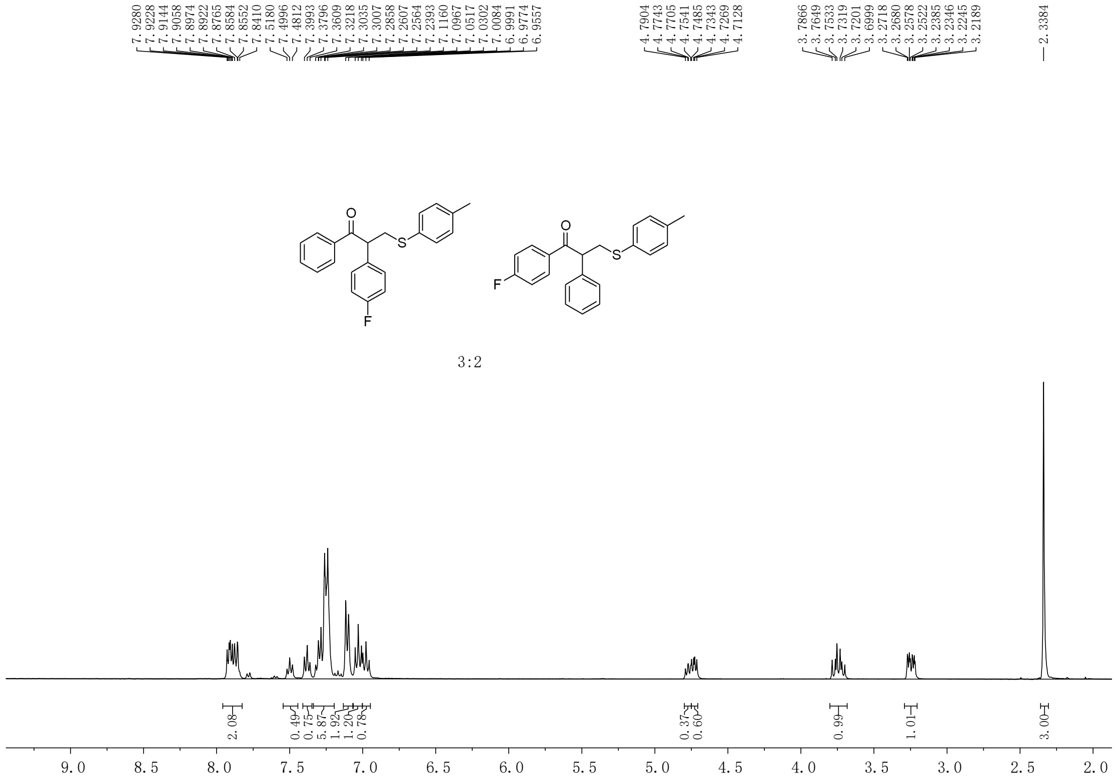

Figure S12: The ¹H NMR (400 MHz) of compounds **3da** and **3da'**

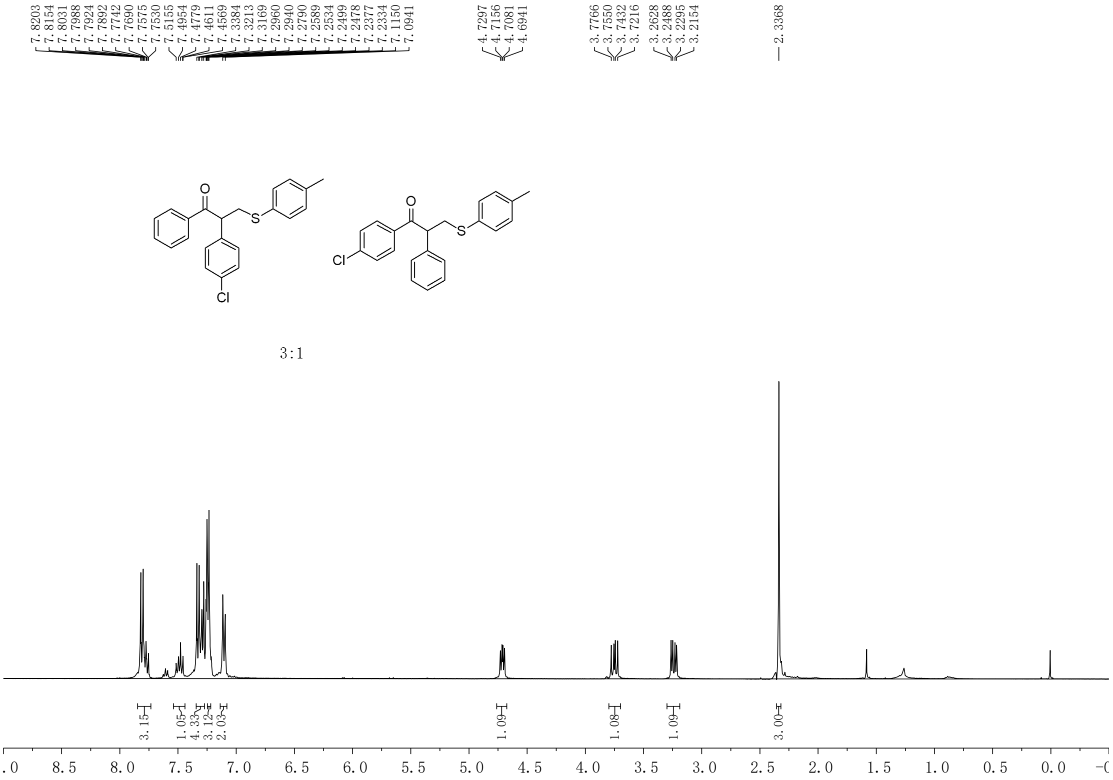

Figure S13: The ^{1}H NMR (400 MHz) of compounds **3ea** and **3ea′**

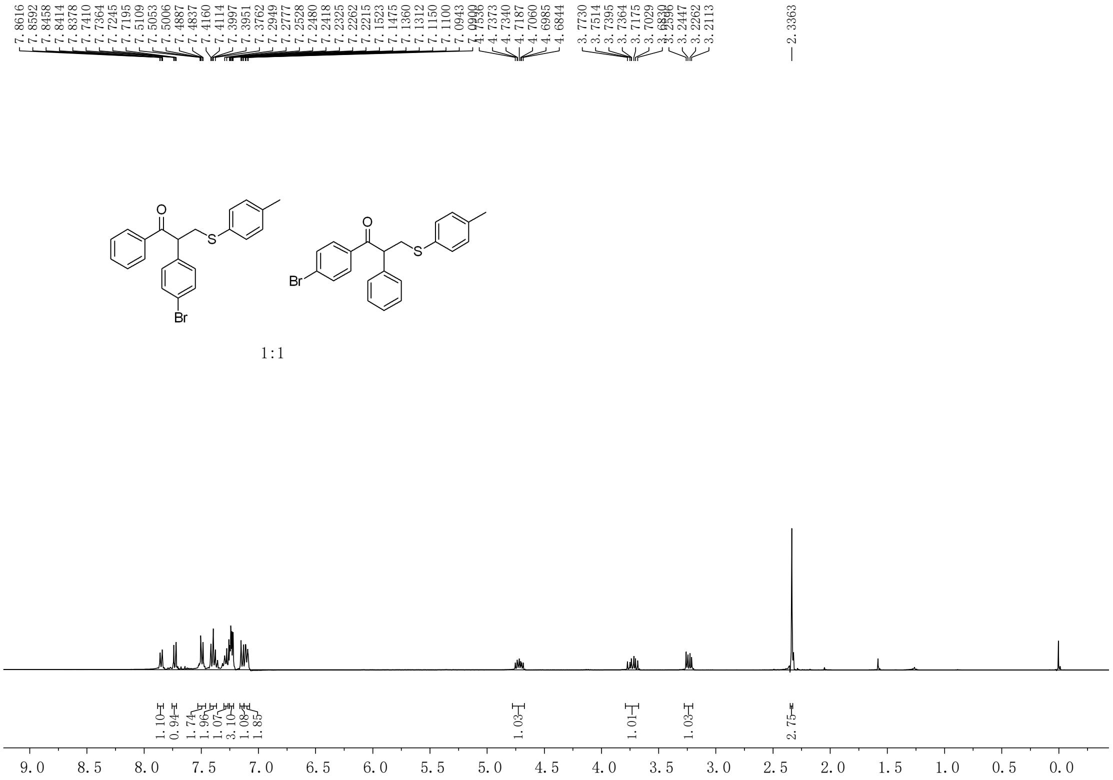

Figure S14: The ^{1}H NMR (400 MHz) of compounds **3fa** and **3fa′**

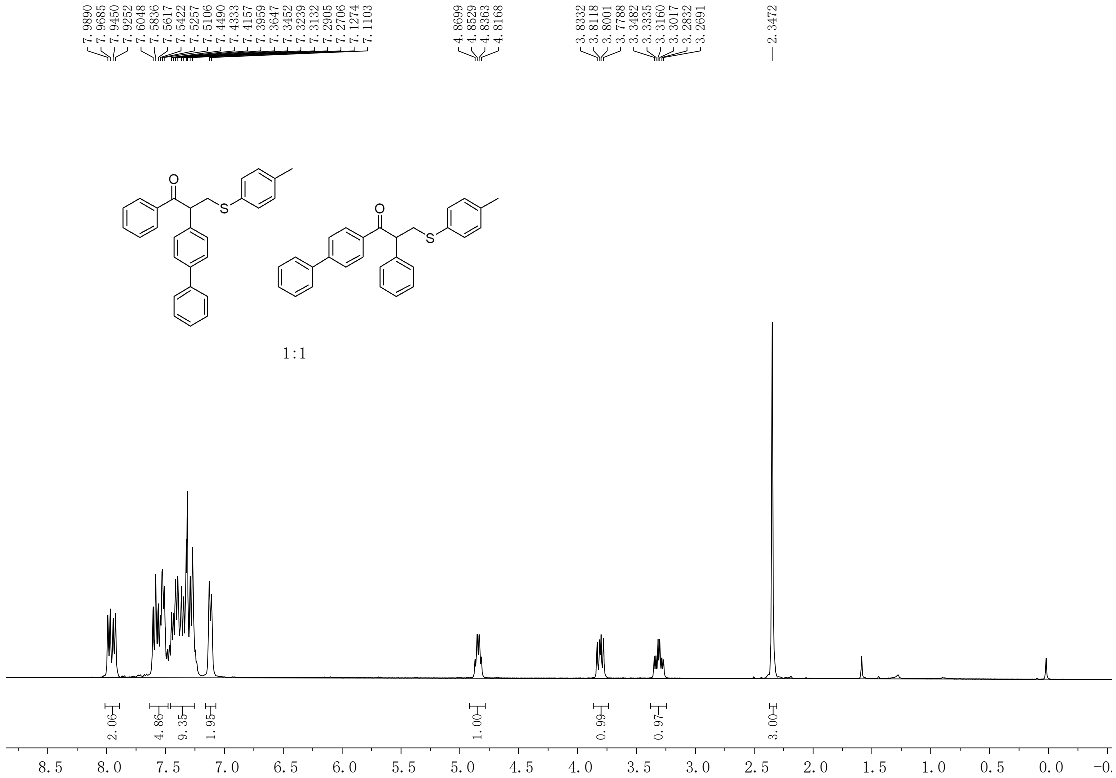

Figure S15: The ¹H NMR (400 MHz) of compounds **3ga** and **3ga'**

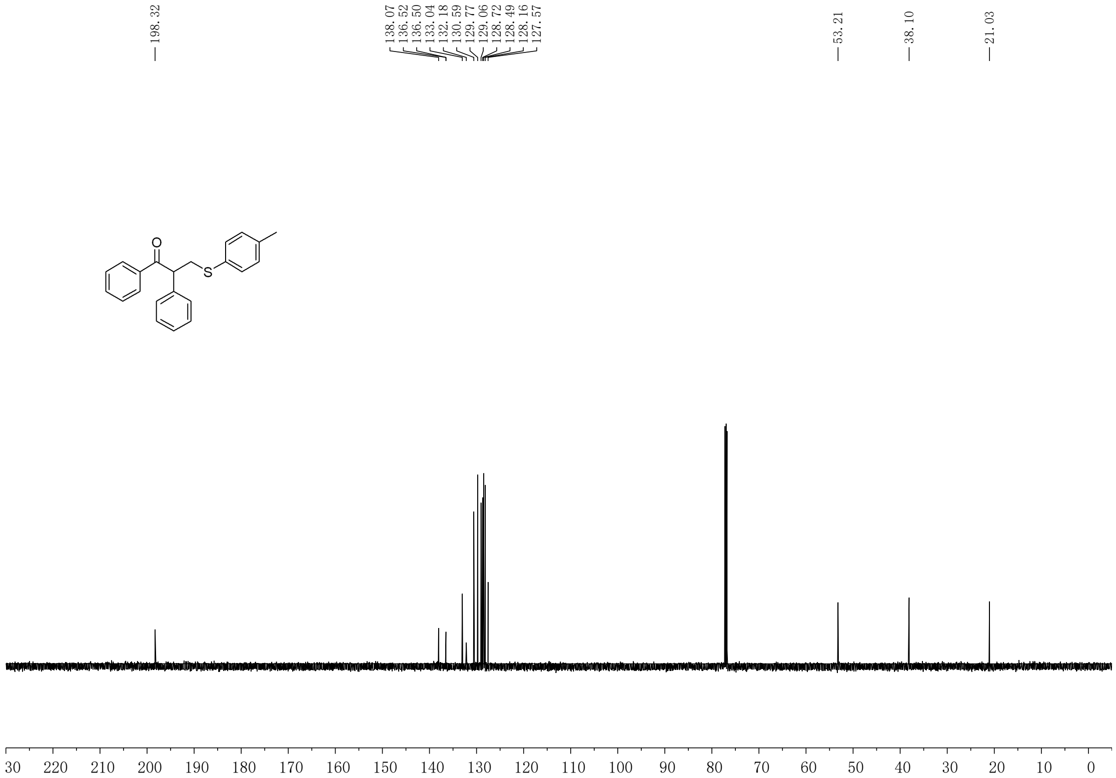

Figure S16: The ¹³C NMR (151 MHz, $CDCl_3$) of compound **3aa**

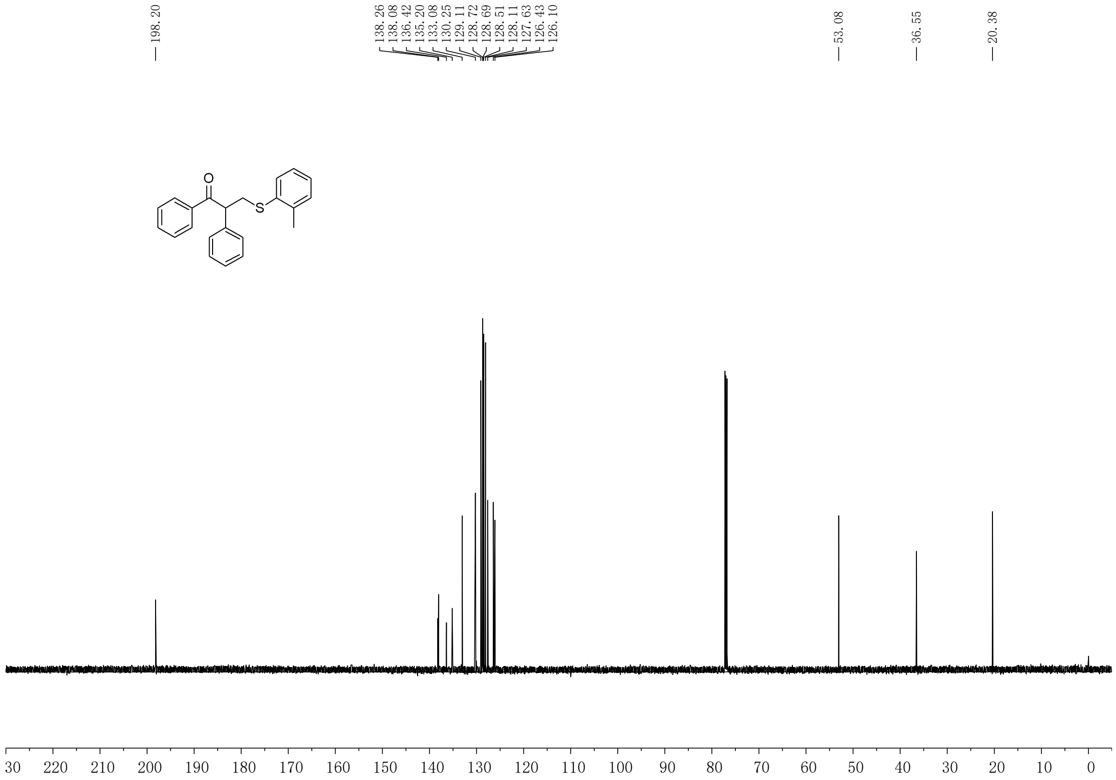

Figure S17: The ^{13}C NMR (151 MHz, $CDCl_3$) of compound **3ab**

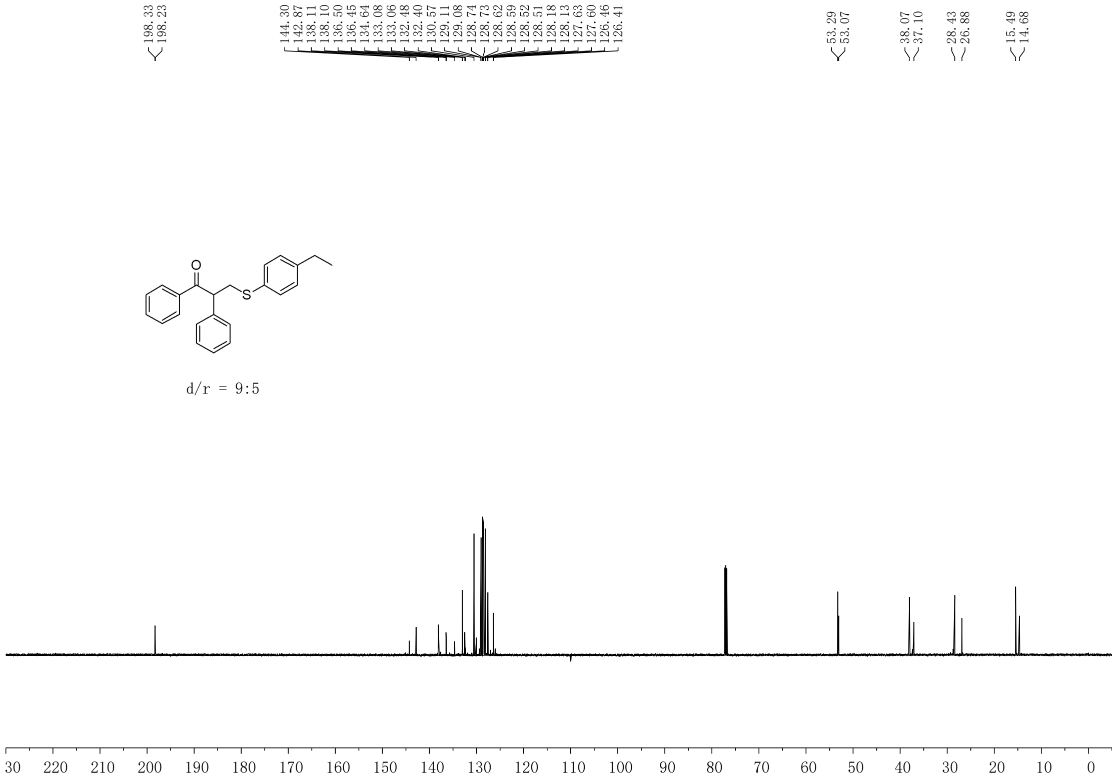

Figure S18: The ^{13}C NMR (151 MHz, $CDCl_3$) of compound **3ac**

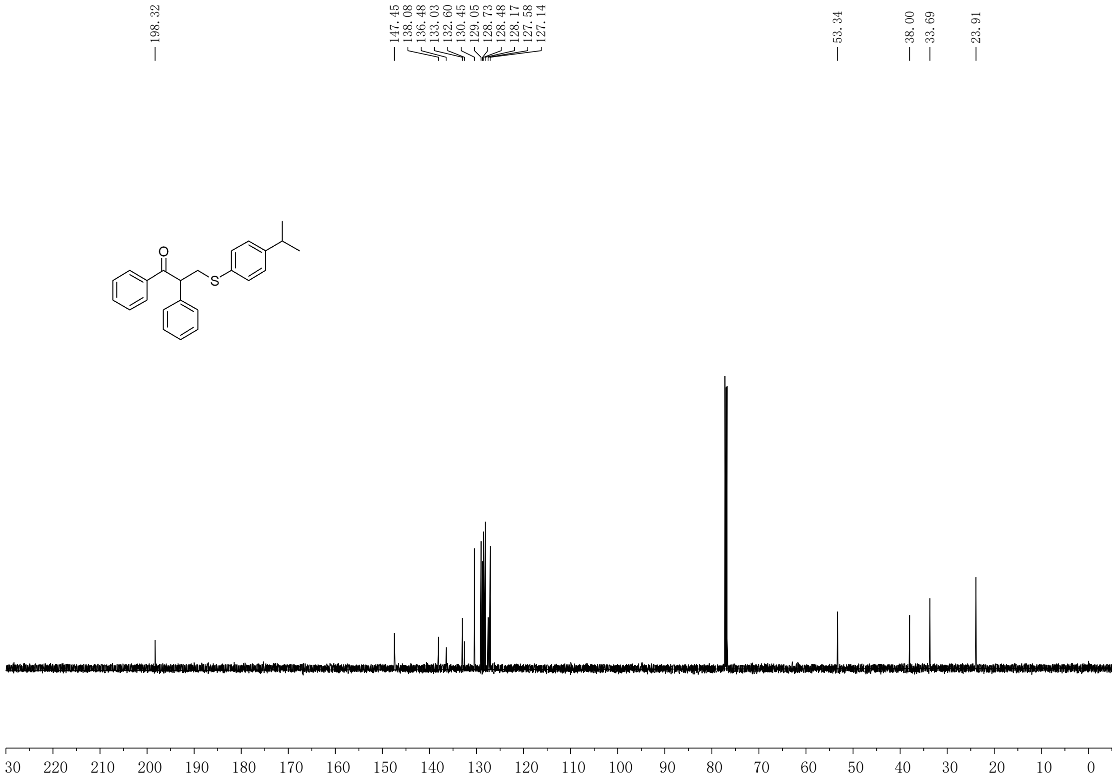

Figure S19: The ^{13}C NMR (151 MHz, $CDCl_3$) of compound **3ad**

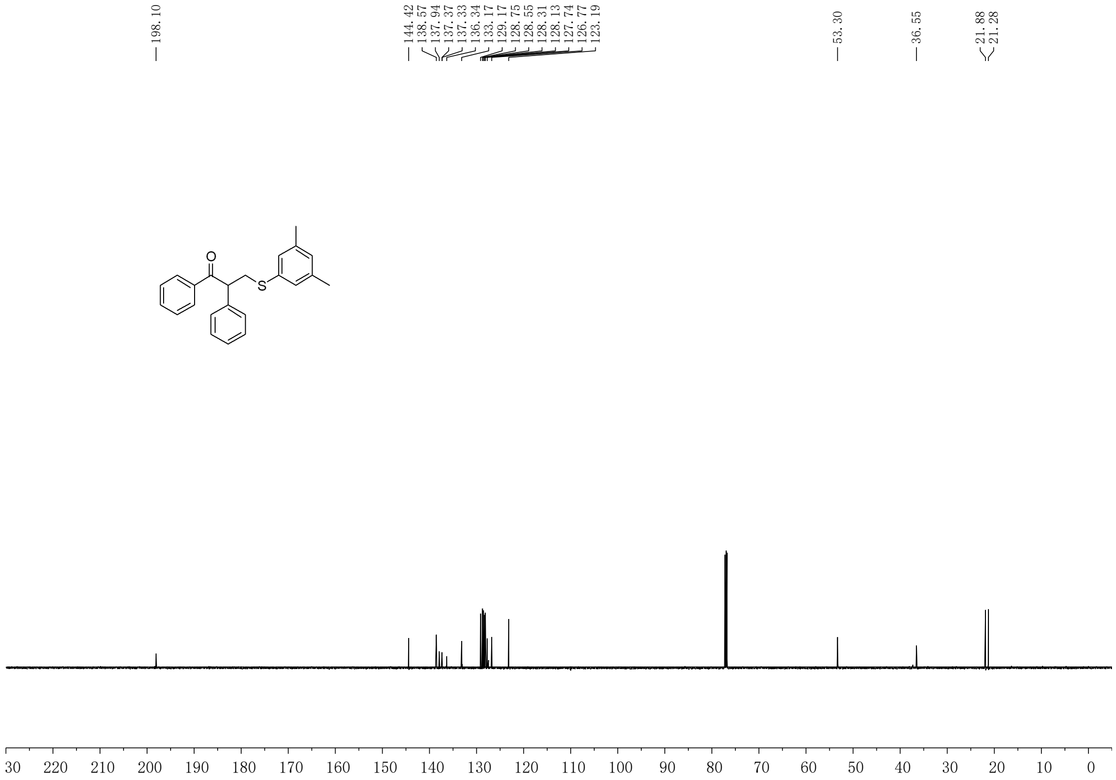

Figure S20: The ^{13}C NMR (151 MHz, $CDCl_3$) of compound **3ae**

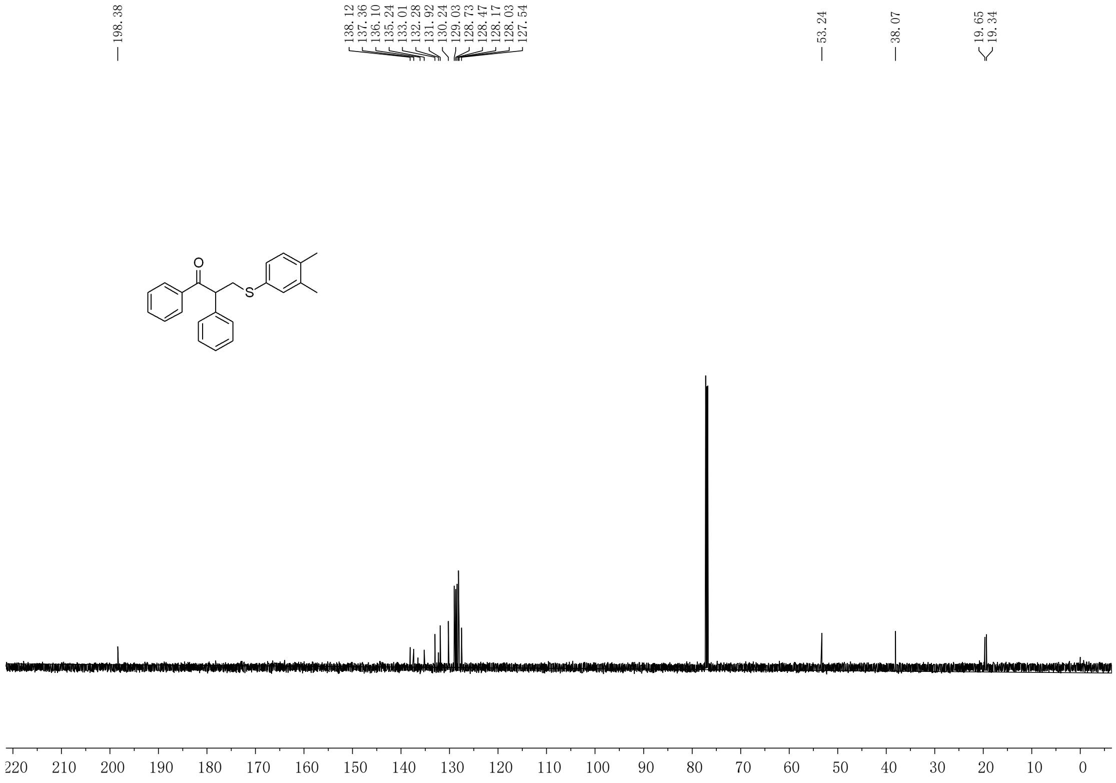

Figure S21: The ^{13}C NMR (151 MHz, $CDCl_3$) of compound **3af**

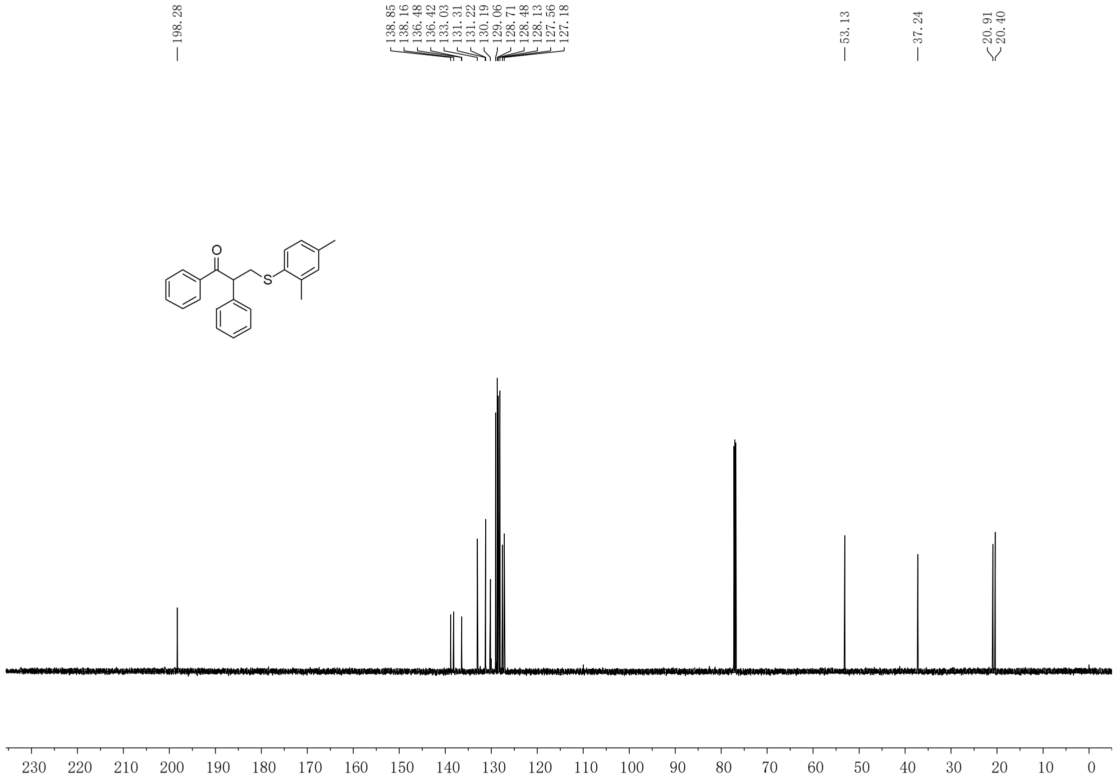

Figure S22: The ^{13}C NMR (151 MHz, $CDCl_3$) of compound **3ag**

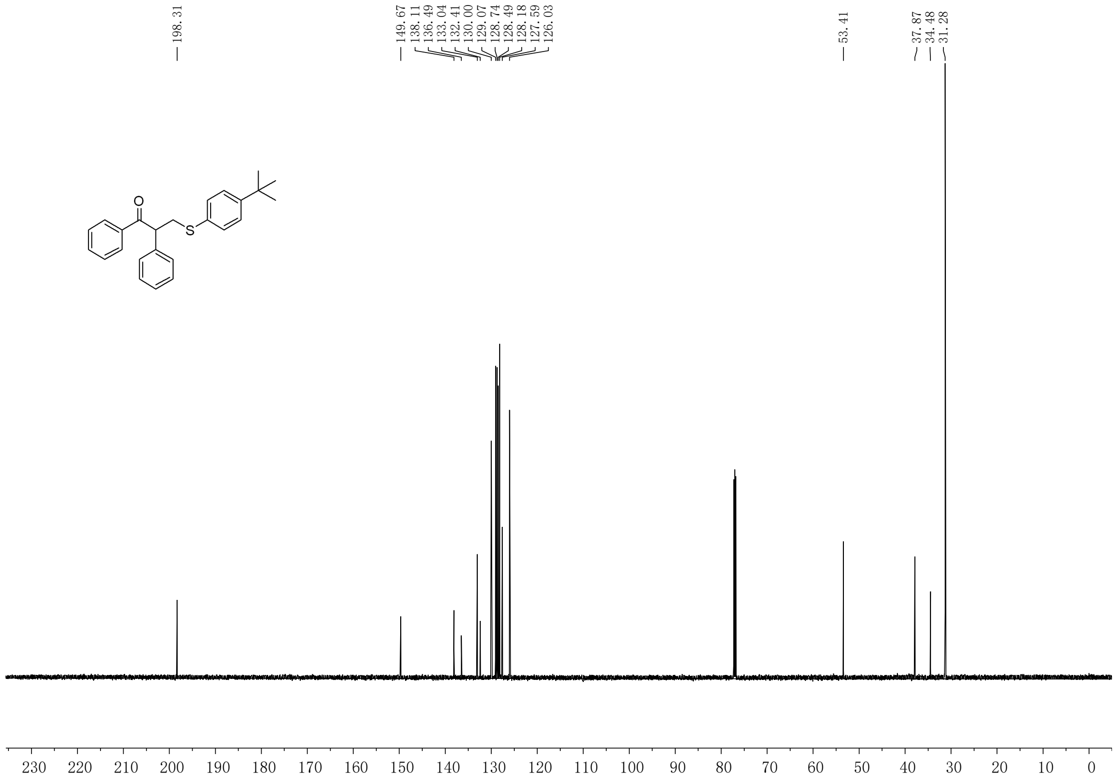

Figure S23: The ^{13}C NMR (151 MHz, $CDCl_3$) of compound **3ah**

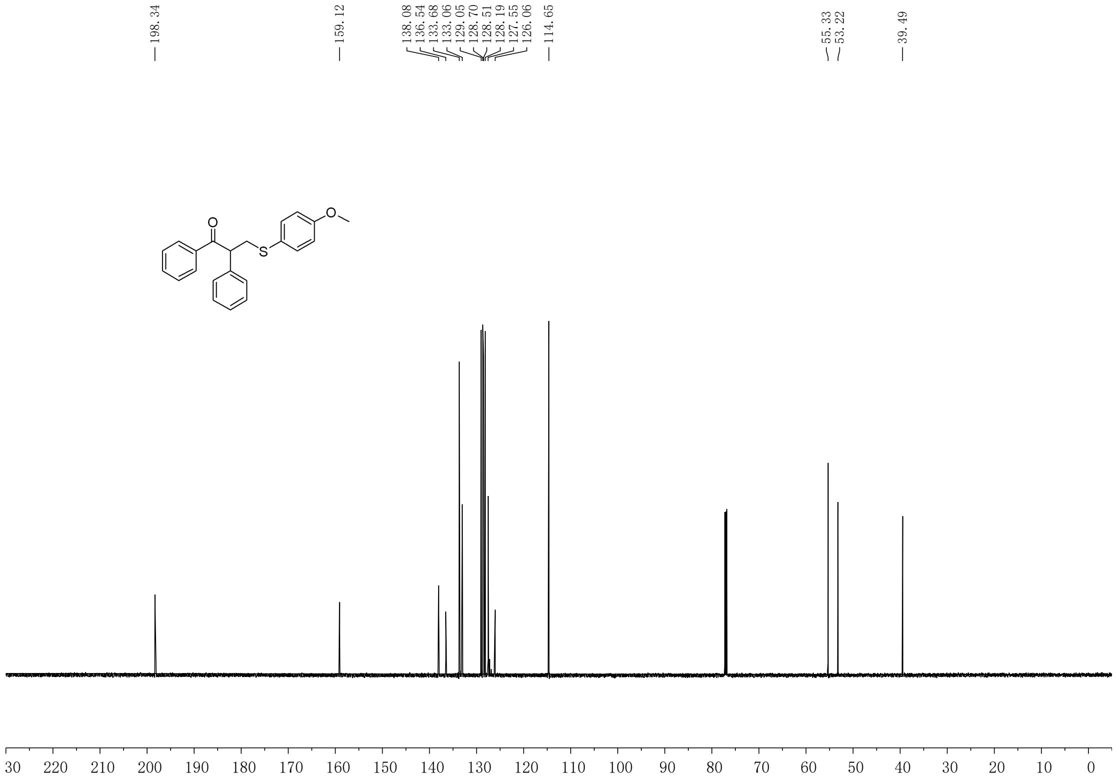

Figure S24: The ^{13}C NMR (151 MHz, $CDCl_3$) of compound **3ai**

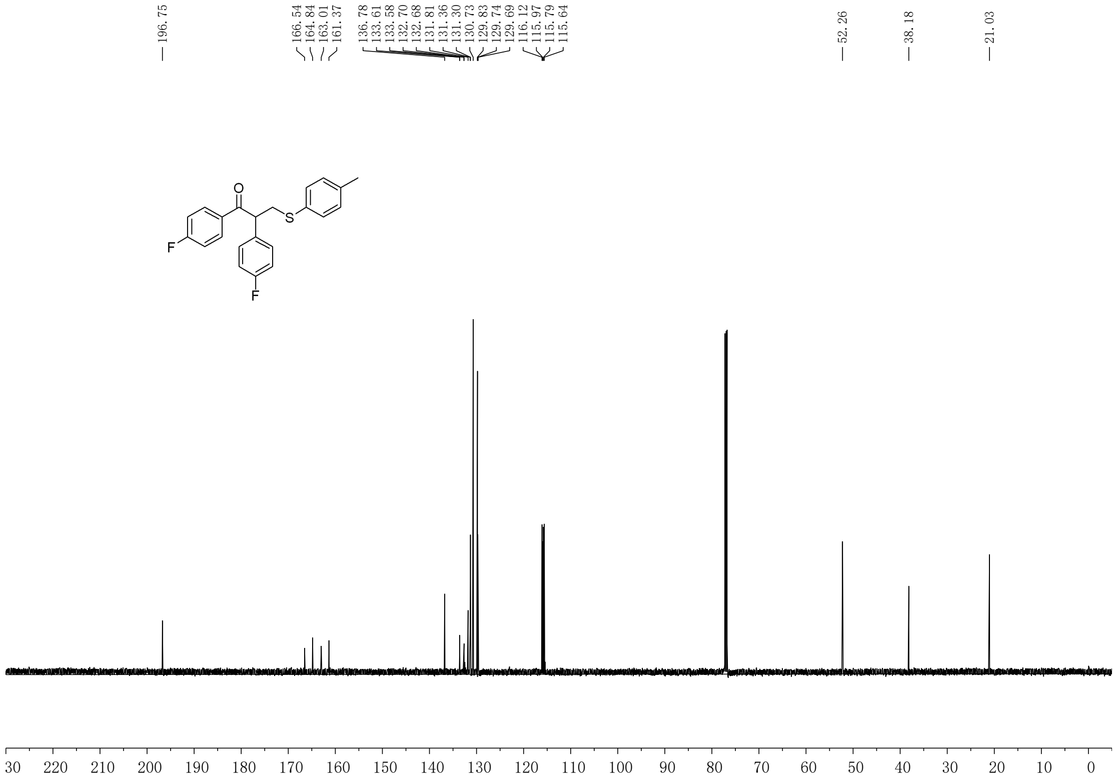

Figure S25: The ¹³C NMR (151 MHz, $CDCl_3$) of compound **3ba**

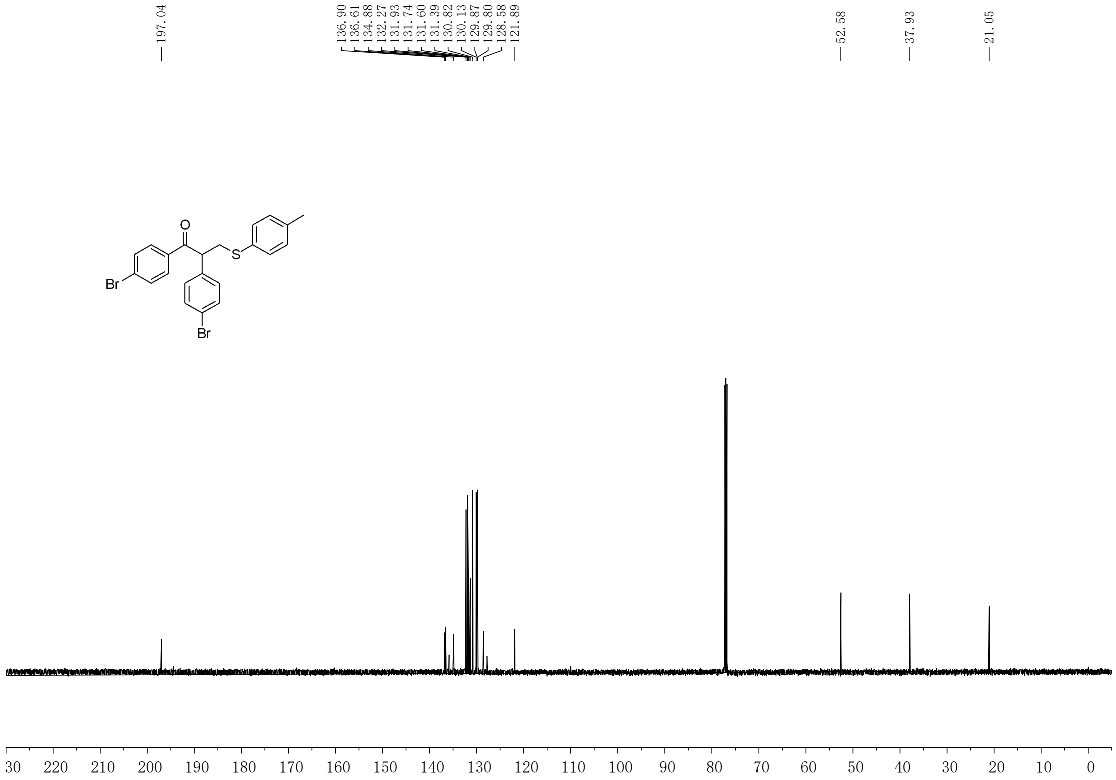

Figure S26: The ¹³C NMR (151 MHz, $CDCl_3$) of compound **3ca**

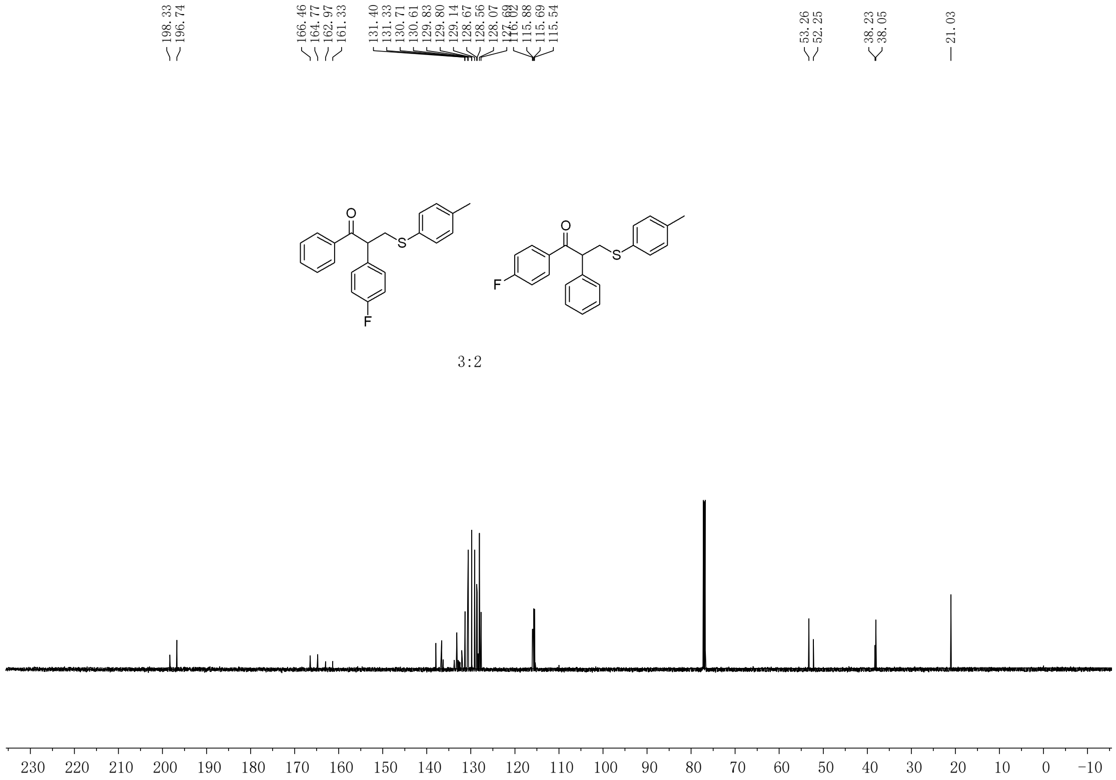

Figure S27: The ^{13}C NMR (151 MHz, $CDCl_3$) of compounds **3da** and **3da′**

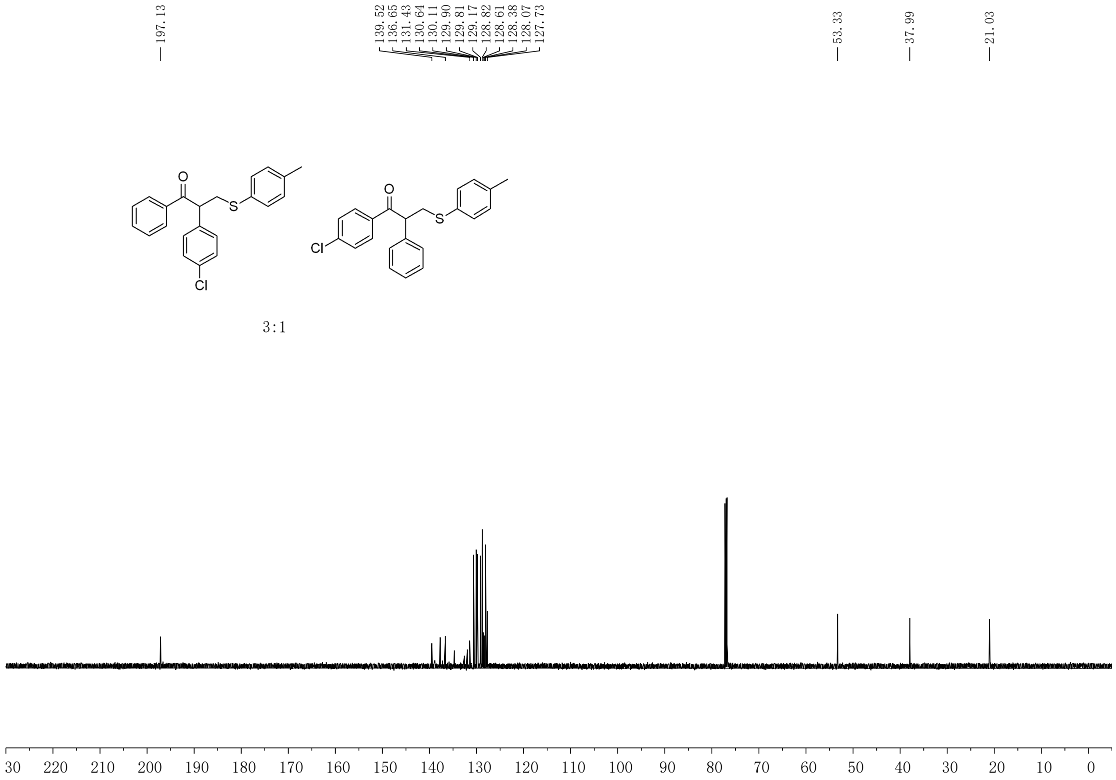

Figure S28: The ^{13}C NMR (151 MHz, $CDCl_3$) of compounds **3ea** and **3ea′**

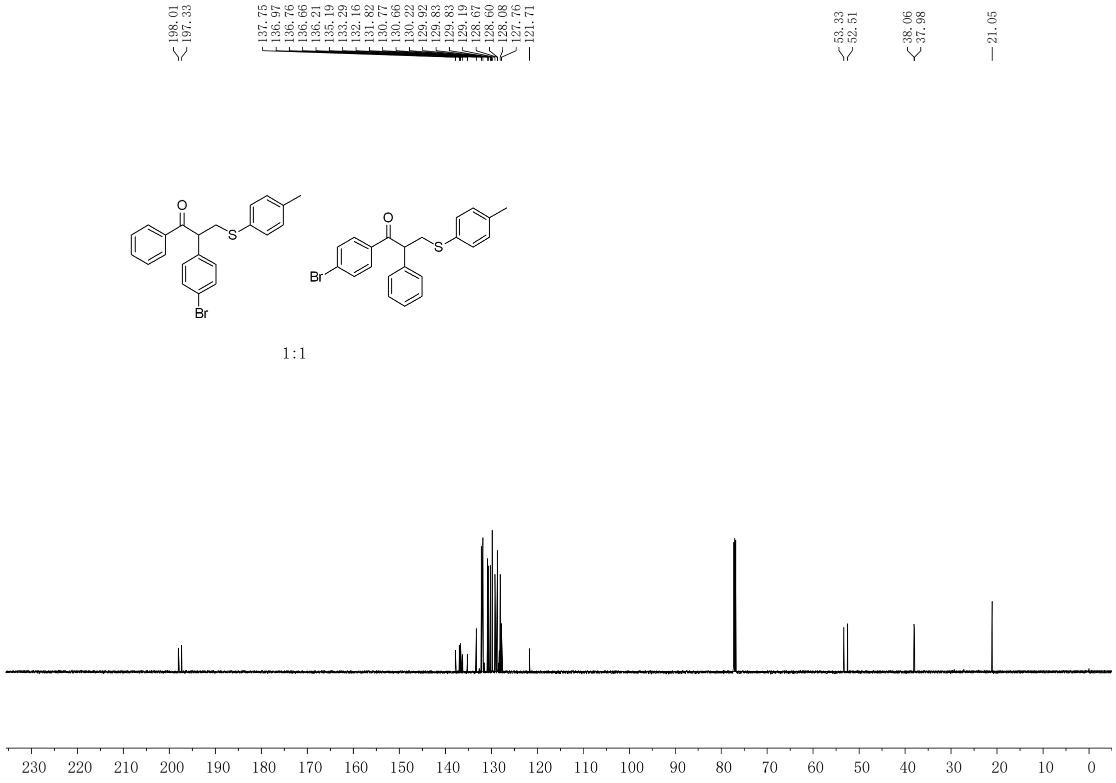

Figure S29: The ^{13}C NMR (151 MHz, $CDCl_3$) of compounds **3fa** and **3fa’**

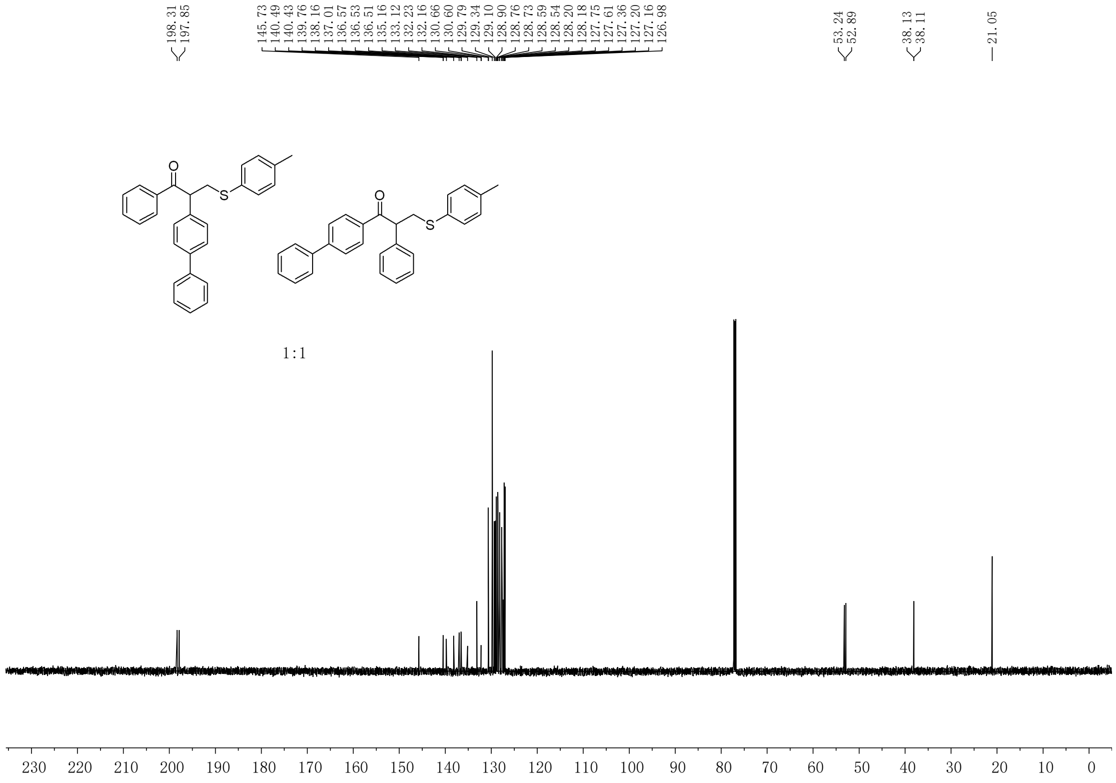

Figure S30: The ^{13}C NMR (151 MHz, $CDCl_3$) of compounds **3ga** and **3ga’**
